# Contribution of *CgPDR1*-Regulated Genes in Enhanced Virulence of Azole-Resistant *Candida glabrata*

**DOI:** 10.1371/journal.pone.0017589

**Published:** 2011-03-09

**Authors:** Sélène Ferrari, Maurizio Sanguinetti, Riccardo Torelli, Brunella Posteraro, Dominique Sanglard

**Affiliations:** 1 Institute of Microbiology, University of Lausanne and University Hospital Center, Lausanne, Switzerland; 2 Institute of Microbiology, Università Cattolica del Sacro Cuore, Rome, Italy; Montana State University, United States of America

## Abstract

In *Candida glabrata*, the transcription factor CgPdr1 is involved in resistance to azole antifungals via upregulation of ATP binding cassette (ABC)-transporter genes including at least *CgCDR1*, *CgCDR2* and *CgSNQ2*. A high diversity of GOF (gain-of-function) mutations in *CgPDR1* exists for the upregulation of ABC-transporters. These mutations enhance *C. glabrata* virulence in animal models, thus indicating that *CgPDR1* might regulate the expression of yet unidentified virulence factors. We hypothesized that CgPdr1-dependent virulence factor(s) should be commonly regulated by all GOF mutations in *CgPDR1*. As deduced from transcript profiling with microarrays, a high number of genes (up to 385) were differentially regulated by a selected number (7) of GOF mutations expressed in the same genetic background. Surprisingly, the transcriptional profiles resulting from expression of GOF mutations showed minimal overlap in co-regulated genes. Only two genes, *CgCDR1* and *PUP1* (for *PDR1* upregulated and encoding a mitochondrial protein), were commonly upregulated by all tested GOFs. While both genes mediated azole resistance, although to different extents, their deletions in an azole-resistant isolate led to a reduction of virulence and decreased tissue burden as compared to clinical parents. As expected from their role in *C. glabrata* virulence, the two genes were expressed as well *in vitro* and *in vivo*. The individual overexpression of these two genes in a *CgPDR1*-independent manner could partially restore phenotypes obtained in clinical isolates. These data therefore demonstrate that at least these two *CgPDR1-*dependent and -upregulated genes contribute to the enhanced virulence of *C. glabrata* that acquired azole resistance.

## Introduction

*Candida glabrata* is a haploid member of Ascomycetes normally not found in the environment but which has rather adapted to conditions found in mammals [1]. Among human fungal pathogens, *C. glabrata* is often reported as the second most prevalent species after *Candida albicans* [2],[3]. *C. glabrata* can cause mucosal and bloodstream infection (BSI) mainly in immuno-compromised hosts. Worldwide, *C. glabrata* accounts for an average 11% of infections caused by *Candida* species, however this proportion varies from 7 to 20% depending on geographical locations [4].

*C. glabrata* infections can be treated with several antifungal agents including amphotericin B, azoles and echinocandins [5], [6]. However, *C. glabrata* can develop antifungal resistance and especially to the class of azole antifungals. Azole resistance surveillance studies have revealed a proportion varying from 10 to 20% of isolates with MIC values reaching clinical breakpoints (e.g. 64 µg/ml for fluconazole, based on CLSI standards). Several countries reported an increase in the proportion of azole-resistant isolates from 2001 to 2007 [4]. *C. glabrata* is also known for exhibiting intrinsically higher azole MIC values than *C. albicans*. For example, the average of fluconazole MIC values of a *C. glabrata* wild type population is near a value of 4 µg/ml, while it is approximately 32-fold lower for *C. albicans* [7], [8]. We and others showed that azole resistance in *C. glabrata* was mediated almost exclusively by enhanced drug efflux and overexpression of multidrug transporters of the ATP Binding Cassette (ABC) transporters. Several genes encoding these transporters were identified including *CgCDR1*, *CgCDR2* (*PDH1*) and *CgSNQ2* [8], [9], [10], [11], [12]. Azole resistance in clinical isolates can be the result of overexpression of single or several transporters [13]. The understanding of regulatory circuits controlling the expression of these genes has progressed in the recent years. A major regulator of these genes, *CgPDR1*, was identified [14], [15]. This gene belongs to the family of zinc finger transcription factors and functionally resembles *PDR1* and *PDR3* from the baker's yeast *Saccharomyces cerevisiae*. Deletion of *CgPDR1* results in a loss of transcriptional control of the major transporters involved in azole resistance and, consequently, decreased resistance to these antifungals [14], [15]. *CgPDR1* exhibits mutations, so called gain-of-function (GOF) mutations, which are responsible for intrinsic high expression of ABC transporters and therefore constitute the molecular basis of azole resistance in *C. glabrata* [13], [14], [15]. One striking feature of GOF mutations is their high diversity among *CgPDR1* alleles from azole-resistant isolates. As many as 67 mutations conferring azole resistance are described up to now [13], [14], [15], [16], [17]. GOF mutations are found within several domains of the transcription factor corresponding to putative functional elements inferred from comparison to the *S. cerevisiae PDR1* and *PDR3* and including the transcriptional activation domain, a regulatory domain and a so-called middle homology region (MHR) which is found in several zinc finger proteins [13], [16].

Not only are GOF mutations in *CgPDR1* important for azole resistance in *C. glabrata* but also for fungal-host interactions. We showed that GOF mutations were associated with enhanced virulence and fitness in animal models of systemic infection [13]. This was unexpected since it is generally accepted that the development of drug resistance in other microbes is usually associated with costs in virulence or fitness. Secondary compensatory mechanisms can however restore the costs of resistance development [18], [19].

In this study we addressed in *C. glabrata* the identification of genes behind the GOF-dependent virulence of *CgPDR1*. Because we rationalized that some genes commonly expressed by GOF mutations could be responsible for this effect, we analysed with transcript profiling analysis *C. glabrata* isolates containing individual GOF mutations but in identical genetic backgrounds. Only two genes (*CgCDR1* and *PUP1*) were identified. We describe here their relevance in the enhanced virulence mediated by *CgPDR1* GOF mutations.

## Results

### Transcriptional analysis of GOF mutations

In a previous study, we reported a high variety of gain-of-function (GOF) mutations in the transcriptional activator *CgPDR1* [13]. These mutations conferred azole resistance through the differentiated upregulation of several ABC transporters including *CgCDR1*, *CgCDR2* and *CgSNQ2*. It is known that *CgPDR1* controls the expression of many other genes, some of which contain a regulatory domain in their promoter matching the PDRE (Pleiotropic Drug Responsive Element) described in *S. cerevisiae* (TCCRYGSR) [14], [16].

We were therefore interested to test whether the differentiated expression pattern observed for a few genes as described earlier [13] could be generalized to the entire transcriptome of *C. glabrata*. In order to achieve this goal, labeled cRNA from mRNA isolated in triplicates from strains containing seven different *CgPDR1* GOF was applied to oligonucleotides custom arrays. The selection of GOFs was based on their occurrence in putative CgPdr1 functional domains including the regulatory domain (L280F, R376W), the MHR (Y584C, T588A) and the activation domain (D1082G, E1083Q). The GOF P822L was also selected since it was previously associated with a strong upregulation of *CgSNQ2* as compared to other ABC-transporters [9]. The format of one-color hybridization was chosen since it allows direct comparisons between any strains. The strains containing the different GOF were obtained by re-introduction of *CgPDR1* alleles at the genomic locus and were described in our previous study [13].

**Figure 1:**
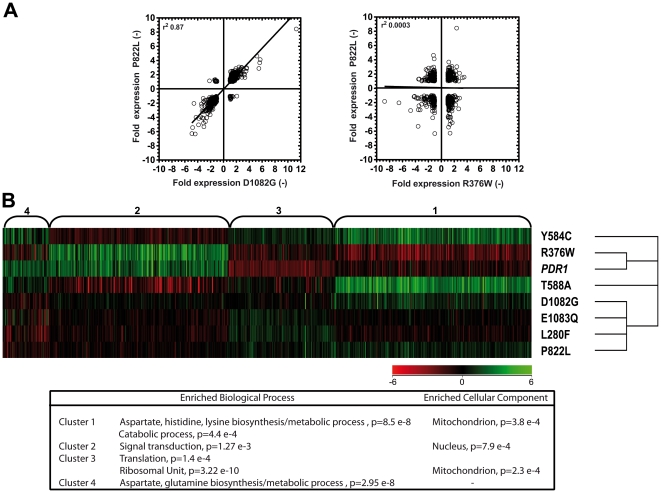
Expression profiles of *C. glabrata* genes regulated by GOFs in *CgPDR1*. **Panel A**: Pairwise comparisons of gene expression changes relative to SFY114 carrying the wild type *CgPDR1* allele. Each data point correlate the same gene expressed in strain SFY116 (P822L GOF) versus strain SFY103 (P1082G GOF) (left side) and in strain SFY116 (P822L GOF) versus strain SFY101 (R376W GOF). For each diagram, r^2^ values are given. **Panel B**: K-means clustering of the normalized expression levels of the 626 genes regulated (≥2-fold) by at least one *CgPDR1* GOF. Clustering was performed with Genespring® GX (parameters: Euclidian distance metric, 100 iterations, 4 clusters). For each cluster, enriched biological function and biological component were determined using GO terms of *S. cerevisiae* homologues. Results are given below the cluster analysis.

As summarized in Table 1, the number of genes differentially regulated (≥2-fold) by individual GOF as compared to the wild type *CgPDR1* ranges from 73 (for the R376W substitution) to 385 (for the T588A substitution) and no GOF regulated a similar number of genes. A total of 626 genes were regulated by at least one GOF (see File S1). The degree of similarity between transcription profiles in the 626 genes regulated could also be estimated with linear regression coefficients, which can establish the extent of gene co-regulation by pairs of separate GOF. As summarized in Table 2, approximately half of r^2^ values from pairwise comparisons were above 0.5 (from 0.54 to 0.87) and thus signified a moderate trend towards the co-regulation of the genes by these GOFs. The highest correlation (r^2^ = 0.87) was observed between expression pattern of GOF D1082G (SFY103) with P822L (SFY116) (Fig. 1A, left side). One GOF (R376W) in SFY101 yielded systematically low r^2^ values with all other GOFs (between 0.0003 and 0.058). Increasing the cut-off for differential regulation to ≥3-fold did not significantly change r^2^ values (data not shown). The expression of genes obtained from GOF P822L (SFY116) and from R376W is shown to illustrate the low level of gene co-regulation between both isolates (Fig. 1A, right side). Taken together, these data support the concept that individual GOF result each in distinct transcription profiles even though the number of GOF analysed is probably only a portion of the entire mutation spectrum.

**Table 1:** Number of *C. glabrata* genes regulated by ≥twofold in *PDR1* GOF mutants as compared to the wild type.

Strain	*CgPDR1* GOF mutation	Genes upregulated	Genes downregulated	Total
SFY101	R376W	27	46	73
SFY103	D1082G	53	77	130
SFY105	T588A	235	150	385
SFY109	E1083Q	58	103	161
SFY111	Y584C	197	132	329
SFY115	L280F	67	132	199
SFY116	P822L	71	89	160

**Table 2:** Correlation coefficients of transcriptional profiles.

GOF in *CgPDR1* allele	L280F	R376W	Y584C	T588A	P822L	D1082G	E1083Q
L280F	1	0.016	0.6111	0.3107	0.7761	0.6979	0.8391
R376W	0.016	1	0.0588	0.0316	0.0003	0.0055	0.0003
Y584C	0.6111	0.0588	1	0.5491	0.7798	0.7012	0.7321
T588A	0.3107	0.0316	0.5491	1	0.4591	0.5596	0.4023
P822L	0.7761	0.0003	0.7798	0.4591	1	0.8704	0.7741
D1082G	0.6979	0.0055	0.7012	0.5596	0.8704	1	0.6984
E1083Q	0.8391	0.0003	0.7321	0.4023	0.7741	0.6984	1

Given the diversity of transcriptional profiles provided by each GOF, the generated transcriptional data were clustered in a separate analysis in order to group sets of genes co-regulated by the different GOFs. Four separated groups were thus identified which were enriched in specific biological processes (Fig. 1B). It is noteworthy that genes from cluster 1 and 4 are enriched in processes related to amino acid metabolism, while others are enriched in signal transduction and protein metabolic processes.

We closely inspected the transcription profiles of two isolates, one carrying the GOF mutation D1082G (SFY103) and the other the mutation P822L (SFY116). This choice was based on the fact that these profiles show the highest correlation (r^2^ = 0.87) and similar numbers of up-and downregulated genes, thus facilitating comparisons (Table 1 and 2). Between the two GOFs, 86 genes were co-regulated (32 upregulated and 54 downregulated) from the total of 626 genes regulated by at least one GOF. The upregulated genes in the SFY103 vs SFY116 comparison showed enrichment for xenobiotic transporter activity (p = 3.7E-3), while the downregulated genes exhibited enrichment in amino acid (arginine, glutamine) biosynthesis processes (p = 5.87E-07 to 2.97E-06). The inspection of conserved motifs in the promoters of upregulated genes yielded the consensus YCCACGGA (Figure S3), which closely resembled the PDRE recognition motif of *PDR1* in *S. cerevisiae* ((TCC[AG][CT]G[G/C][A/G]) [20]. These data are therefore consistent with the role of *CgPDR1* in the regulation of genes by the GOF mutations D1082G and P822L.

To determine whether the expression of genes differentially regulated by the GOF mutations was also affected by the absence of *PDR1*, we analysed the expression profile of the *pdr1*Δ strain SFY92. A total of 247 genes were differentially regulated (≥2-fold) in strain SFY92 as compared to SFY114 (containing the *CgPDR1* wild type allele). Analysis of the 99 downregulated genes showed that one third of these genes encode for proteins predicted to be localized in the mitochondria. Moreover, enrichment of specific biological processes (oxidation-reduction, ATP synthesis coupled to electron transport chain, cellular respiration) was observed (File S2). Consistent with these observations is that *PDR1* and *PDR3* in *S. cerevisiae* are known to participate into the mitochondria-nucleus signalling pathway [21], which may also be applied to *CgPDR1*. Finally, 121 genes were differentially regulated not only in absence of *PDR1* but also in the presence of GOF mutations, indicating that these genes might represent the basal set of *PDR1*-dependent genes.

### Virulence determinants in *C. glabrata*

**Figure 2:**
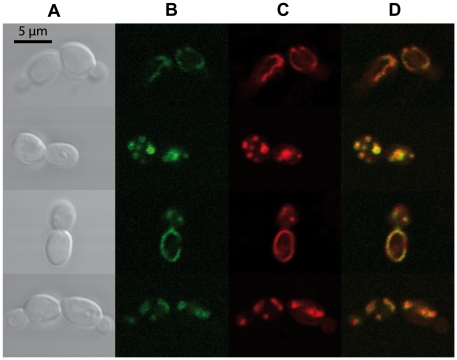
Localization of Pup1p in mitochondria. SFY174 cells expressing the Pup1p-GFP fusion protein were stained with Mitotracker Red and fixed as described in Materials and Methods. **Panel A**: Nomarski images of the cells; **panel B**: Pup1p-GFP; **panel C**: mitochondria stained with Mitotracker Red; **panel D**: merging of B and C. Four individual images are shown. Bar, 5 µm.

We reported that GOF mutations analysed here by transcriptional profiling in *C. glabrata* not only resulted in azole resistance but also in enhanced virulence and fitness in a mice model of infection [13]. We reasoned that enhanced virulence could be due to specific genes commonly regulated by all *CgPDR1* GOFs, given that this phenotype was shared by all these mutations. Our current analysis revealed that no gene was commonly downregulated and only two genes were commonly upregulated by at least two-fold by all GOFs, i.e. *CgCDR1*, the well-known ABC-transporter involved in azole resistance, and the ORF CAGL0M12947g, which we named *PUP1* (for *PDR1* UPregulated gene) in the present study. This gene is highly similar to *YIL077c*, a gene encoding a protein of unknown function thought be located in the mitochondria. We tested this hypothesis in *C. glabrata* by the expression of a GFP-tagged version of *PUP1* in the azole-resistant clinical isolate DSY565. As shown in Fig. 2, the GFP signal could be detected in DSY565. Moreover, Mitotracker Red staining (Panel C), which specifically reveals mitochondrial punctuate and tubular structures, co-localized with GFP signals of Pup1-GFP. These results therefore confirmed that *PUP1* encodes a mitochondrial protein.

**Figure 3:**
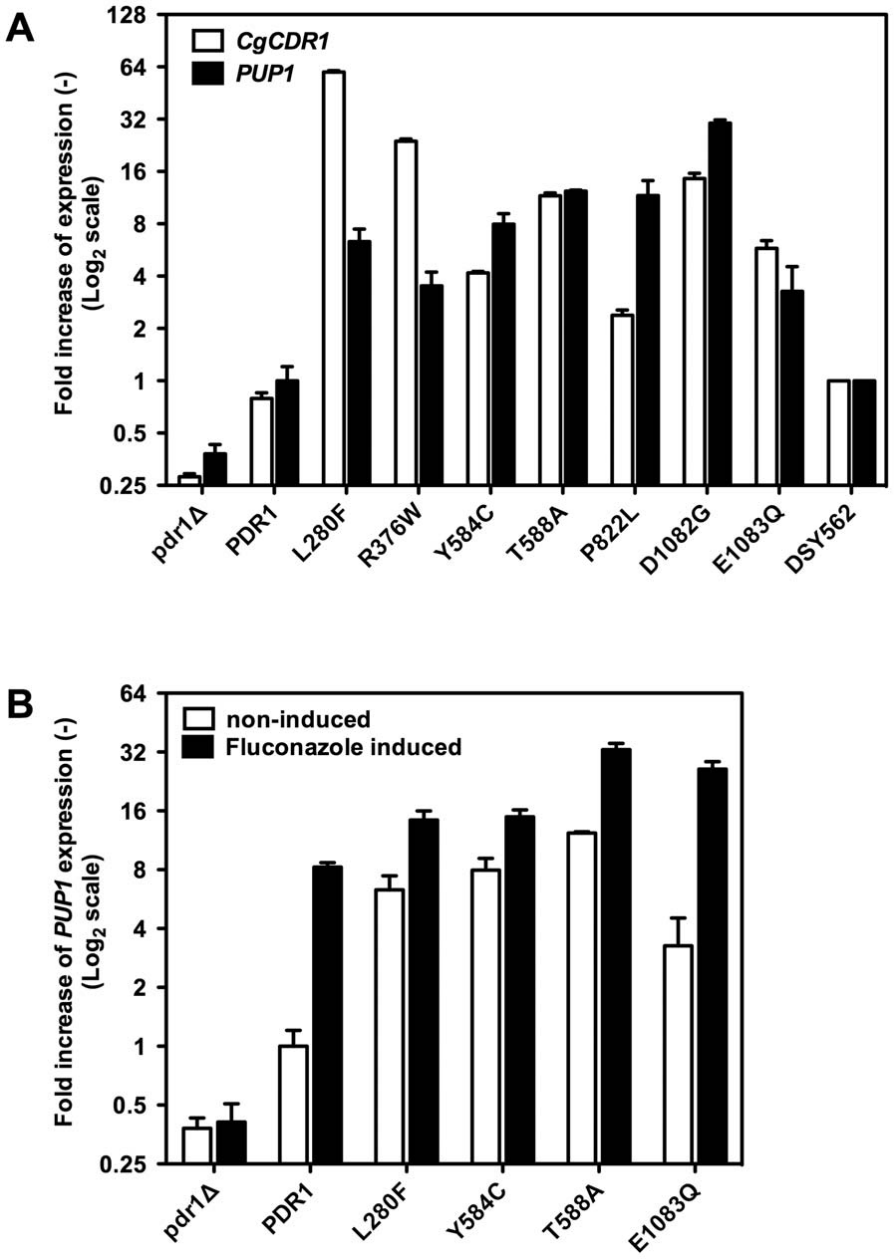
Expression of *CgCDR1* and *PUP1 in vitro*. **Panel A**: Expression of *CgCDR1* and *PUP1* in isolates containing distinct *CgPDR1* alleles. **Panel B**: Expression of *PUP1* after exposure to 256 µg ml^−1^ fluconazole during 150 min. Quantification was performed by qRT-PCR. The values are averages of three separate experiments and represent the increase in gene expression relative to DSY562 (set at 1.00). Strains were constructed from a *pdr1*Δ mutant and were named by the re-introduced GOF mutation or wild type *CgPDR1* allele. The indicated names correspond to the following strains: *pdr1*Δ: SFY92, *PDR1*: SFY114, L280F: SFY115, R376W: SFY101, Y584C: SFY111, T588A: SFY105, P822L: SFY116, D1082G: SFY103, E1083Q: SFY109).

**Figure 4:**
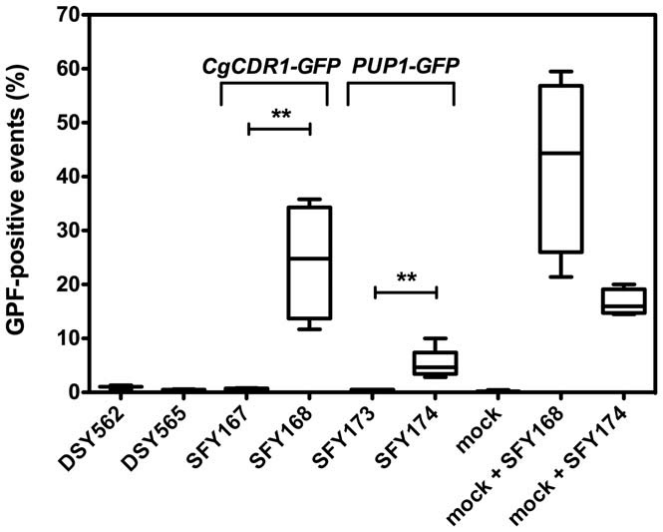
Expression of *CgCDR1* and *PUP1 in vivo*. Flow cytometry analysis of GFP-positive yeast cells was performed from mice kidneys. Groups of 4 mice were injected intravenously with 4×10^7^ CFU of *C. glabrata* strains. Mice were sacrificed at day 7 post-infection. Results are expressed as percents of GFP-positive events in FACS and represent values recorded separately for each mouse. Asterisks indicate statistically significant differences (*: *P*<0.05; **: *P*<0.01, ***: *P*<0.001). Strains SFY167 and SFY168 express the *CgCDR1p-3xGFP* construct and are derived from DSY562 and DSY565, respectively. Strains SFY173 and SFY174 express the *PUP1-3xGFP* construct and are derived from DSY562 and DSY565, respectively. As controls, kidneys of uninfected mice (mock) were analyzed alone or mixed with 1×10^7^ cells of SFY168 or SFY174 grown in YEPD.

*CgCDR1* and *PUP1* are overexpressed by all GOFs and therefore they may constitute good candidates to be responsible for the enhanced virulence observed in animal models. *In vitro*, both genes were dependent on the presence of *CgPDR1* (Fig. 3A). Moreover, *PUP1* contains two PDREs in its promoter (−770 to −763: TCCACGGA; −740 to −733: TCCGTGGA) and *PUP1* expression was inducible by fluconazole (Fig. 3B) similarly to *CgCDR1*. Because they might be important for the enhanced virulence phenotype, these genes should also be expressed *in vivo*. We tested this hypothesis by injecting mice with strains expressing the GFP under the control of the *CgCDR1* promoter or fused to the *PUP1* ORF. Kidneys homogenates were recovered and analysed by flow cytometry to identify GFP-positive yeast cells. As shown in Fig. 4, GFP could be easily detected in the azole-resistant background DSY565 (SFY168) that expresses GFP under the control of the *CgCDR1* promoter. This was not the case in the DSY562 background (SFY167), where GFP expression driven by the *CgCDR1* promoter is low. Similarly, GFP signals in yeast cells expressing the GFP-tagged *PUP1* were detectable in the DSY565 background (SFY174), but not in the DSY562 background (SFY173). The results are consistent with the *in vitro* experiments performed with both GFP-tagged genes and thus indicate that *CgCDR1* and *PUP1* are overexpressed by *CgPDR1* GOF both *in vitro* and *in vivo*.

**Figure 5:**
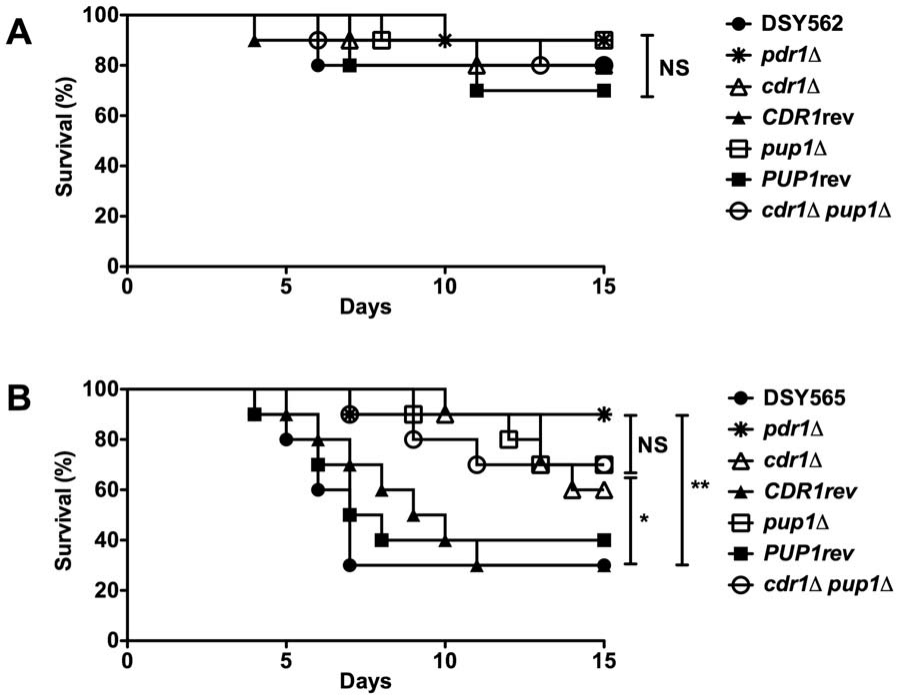
Virulence of *C. glabrata* is dependent on *CgCDR1* and *PUP1*. Survival curves of mice infected with DSY562 (panel A) and DSY565 (panel B) and derived mutants. Statistical differences were performed using the Log-rank Mantel-Cox test (Prism 5.0) by comparing survival curves of mice infected by the parental strains (DSY562 or DSY565) and by other strains as indicated. Asterisks indicate statistically significant differences (*: *P*<0.05; **: *P*<0.01, ***: *P*<0.001). NS indicates no significance (*P*>0.05). For strains derived from DSY562, the indicated names correspond to the following strains: *pdr1*Δ: SFY92, *cdr1*Δ: SFY148, *CDR1*rev: SFY161, *pup1*Δ: SFY150, *PUP1*rev: SFY159, *cdr1*Δ, *pup1*Δ: SFY152. For strains derived from DSY565, the indicated names correspond to the following strains: *pdr1*Δ: SFY94, *cdr1*Δ: SFY149, *CDR1*rev: SFY162, *pup1*Δ: SFY151, *PUP1*rev: SFY160, *cdr1*Δ, *pup1*Δ: SFY153.

To test whether *CgCDR1* and *PUP1* were involved in *C. glabrata* virulence, mutants were constructed in both the genetic backgrounds of DSY562 and DSY565 resulting in strains SFY148 and SFY149 (*CgCDR1* mutants) and SFY150 and SFY151 (*PUP1* mutants), respectively. The deletion of the genes was verified by Southern analysis (see Figure S2). The constructed mutants were next injected intravenously in mice and mice survival was recorded over time. In this model, mice are immuno-compromised by cyclophosphamide treatment. In general, deletion of *CgCDR1* and *PUP1* in DSY562 background had no significant effects as compared to the azole-susceptible isolate DSY562 (Fig. 5). On the contrary, the deletion of *CgCDR1* or *PUP1* in DSY565 resulted in a significant decrease in virulence as compared to the wild type (SFY149 vs DSY565: p = 0.04; SFY151 vs DSY565: p = 0.02). Deleting both genes from DSY565 (SFY170) had a no significant effect as compared to single mutants. In addition, revertant isolates, SFY160 and SFY162, restored *PUP1* and *CgCDR1* expression, respectively, and the phenotype of the wild type parent.

**Figure 6:**
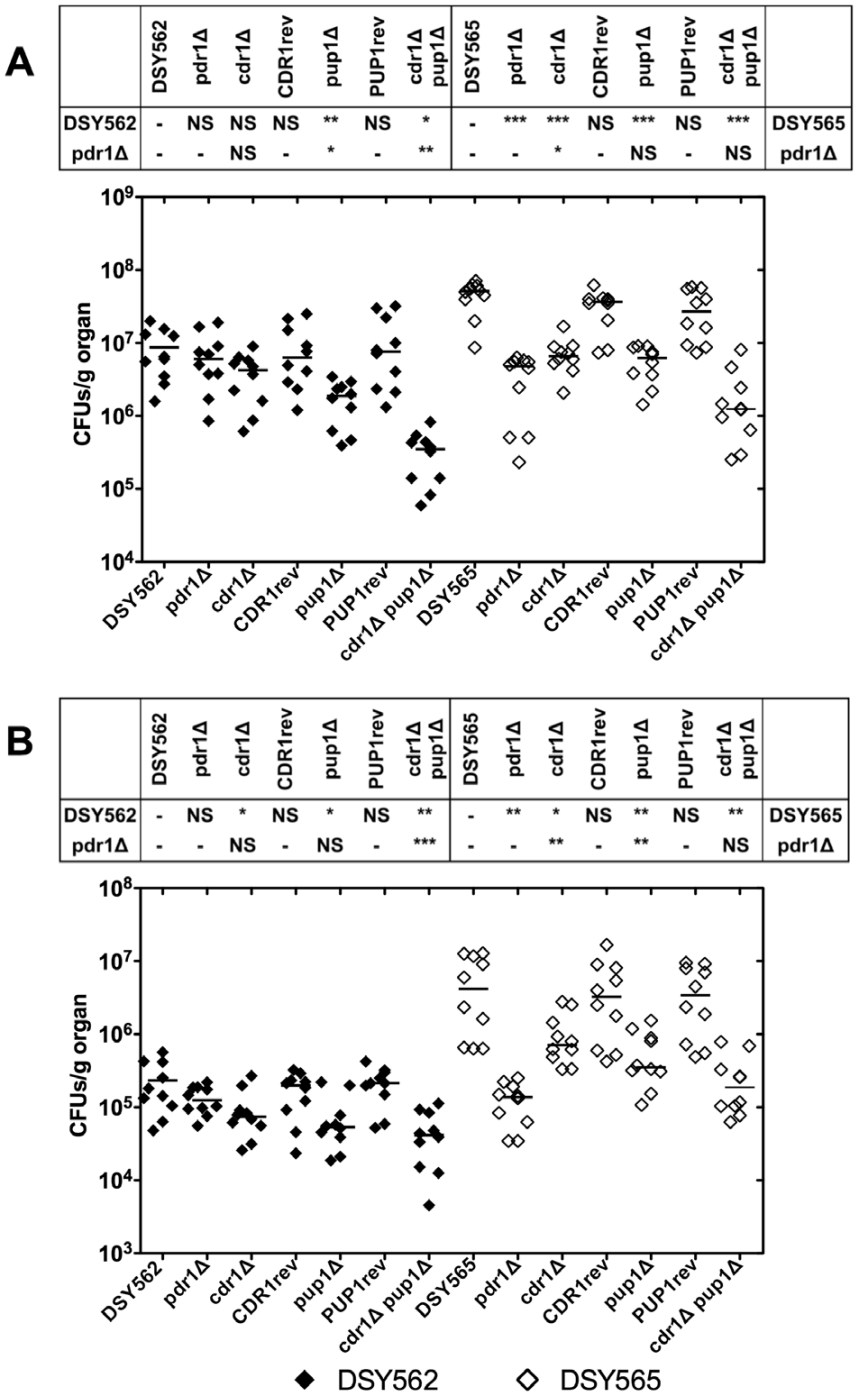
*C. glabrata* tissue burdens in murine infection models. Fungal tissue burdens in kidneys (panel A) and spleen (panel B) from BALB/c mice infected intravenously with 4×10^7^ viable cells of *C. glabrata* strains. Mice were sacrificed at day 7 post-infection. Results are expressed as CFUs per gram of tissue and represent values recorded separately for each of the ten mice. Geometric means are indicated by horizontal bars. Statistical comparisons are summarized above each panel. Asterisks indicate statistically significant differences (*: *P*<0.05; **: *P*<0.01, ***: *P*<0.001). NS indicates no significance (*P*>0.05). The symbol ‘-’ indicates that the statistical comparison was not performed. Statistical differences were determined using the non-parametric Wilcoxon Rank sum tests (Prism 5.0). The origin of each strain is indicated; strain background (DSY562 and DSY565) is indicated by filled or empty symbols, respectively. See legend of Fig. 5 for strain designations.

Tissue burdens were assessed at day 7 post infection and are shown in Fig. 6. In this model, mice are immunocompetent and the endpoint measurement is not mice survival but rather tissue colonization by the infection agent. CFU values were compared with each other. In isolates derived from DSY562, it is interesting to observe that the deletion of *PUP1*, even if it did not result in a decrease of mice survival as compared to the wild type, significantly decreased kidney colonization. This decrease was compensated by the reintroduction of *PUP1* in the mutant (SFY160). This decrease was even more pronounced in the absence of both *PUP1* and *CgCDR1* (SFY169). In isolates derived from DSY565, the individual deletion of *CgCDR1* and *PUP1* (SFY150 and SFY151) decreased CFU counts in a significant manner as compared to the parent strain, a change which was restored by revertants of the corresponding genes. The double deletion of *PUP1* and *CgCDR1* decreased CFU counts in comparison to all other conditions, as observed from DSY5652-derived strains, indicating that *CgCDR1* and *PUP1* deletions have an additive effect on tissue colonization.

Taken together, these results strongly suggest that *CgCDR1* and *PUP1*, two genes upregulated by all *CgPDR1* GOF mutations, are important for the enhanced virulence phenotype observed in the azole-resistant isolate DSY565. Decreased virulence from DSY565-derived strains was associated with decreased tissue colonization and mutant phenotypes could be reverted by the corresponding wild type genes.

### Overexpression of *CgCDR1* and *PUP1* in a *CgPDR1*-independent manner

**Figure 7:**
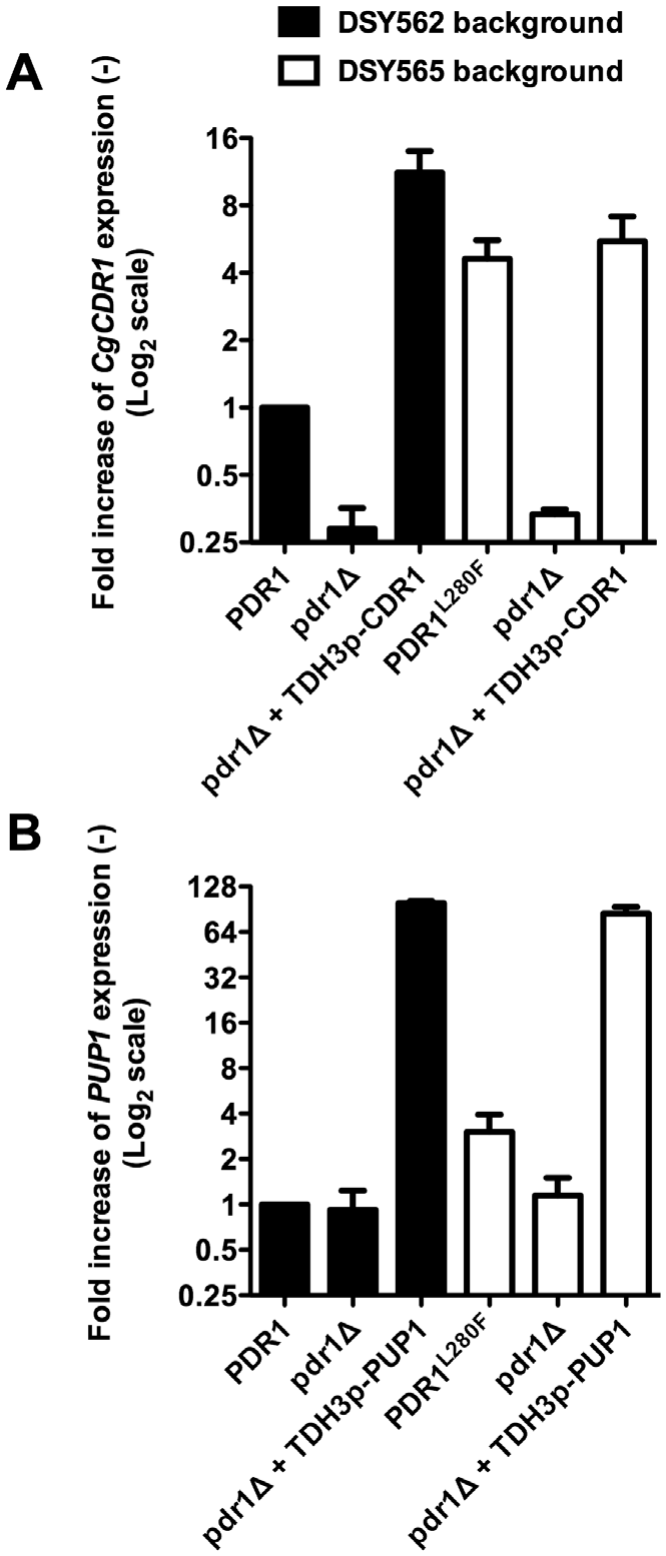
Overexpression of *CgCDR1* and *PUP1* in a *CgPDR1*-independent manner. **Panel A**: *TDH3*-dependent expression of *CgCDR1*. **Panel B**: *TDH3*-dependent expression of *PUP1*. Quantification was performed by qRT-PCR. The values are averages of three separate experiments and represent the increase in gene expression relative to SFY196 (set at 1.00). Strains derived from DSY562 are represented by black bars and the indicated names correspond to the following strains: *PDR1*: SFY196, *pdr1*Δ: SFY198, *pdr1*Δ+*TDH3p-CDR1*: SFY200, *pdr1*Δ+*TDH3p-PUP1*: SFY202. Strains derived from DSY565 are represented by white bars and the indicated names correspond to the following strains: *PDR1^L280F^*: SFY197, *pdr1*Δ: SFY199, *pdr1*Δ+*TDH3p-CDR1*: SFY201, *pdr1*Δ+*TDH3p-PUP1*: SFY203.

The overexpression of *CgCDR1* and *PUP1* is under the control of *CgPDR1* in *C. glabrata*. We showed in the above experiments that both *CgCDR1* and *PUP1* have impact on *C. glabrata* virulence. However, these experiments were carried out in the background of a functional *CgPDR1* and it is possible that other *CgPDR1*-dependent factors contribute to enhanced virulence of azole-resistant isolates. We therefore expressed *CgCDR1* and *PUP1* with a strong constitutive promoter (*TDH3*) in the background of a *CgPDR1* deletion strainto avoid interference with such factors. As observed in Fig. 7, the engineered strains could overexpress both genes at different levels but still to higher levels than *pdr1*Δ mutants. *CgCDR1* levels were approximately equal to those measured in the azole-resistant isolate DSY565 (Fig. 7A), while *PUP1* levels were higher (approx. 20-fold) when expressed under the control of the *TDH3* promoter than the native promoter (Fig. 7B). However, both genes were expressed to similar levels in DSY562 and DSY565 as expected from the constitutive expression from the *TDH3* promoter. Azole MICs strains were 32 µg/ml fluconazole in strains overexpressing *CgCDR1* via the *TDH3* promoter, while the fluconazole MICs were almost identical to the parent strains when *PUP1* was overexpressed (1–2 µg/ml, Table 3), indicating that *CgCDR1* is the major mediator of azole resistance in our strains.

**Table 3:** Fluconazole susceptibilities of *CgCDR1*, *PUP1* and *CgPDR1* mutant strains derived from strains DSY562 and DSY565.

	Fluconazole MIC (µg ml^−1^)^a^								
	Wild type	*pdr1*Δ	*cdr1*Δ	*pup1*Δ	*cdr1*Δ*+CgCDR1*	*pup1*Δ*+PUP1*	*cdr1*Δ *pup1*Δ	*pdr1*Δ*+TDH3p-CDR1*	*pdr1*Δ*+TDH3p-PUP1*
DSY562	4	1	1	2	4	4	1	32	1
DSY565	128	1	4	64	128	128	2	32	2

a MICs were determined by the broth microdilution method according to EUCAST document EDef 7.1 [42].

**Figure 8:**
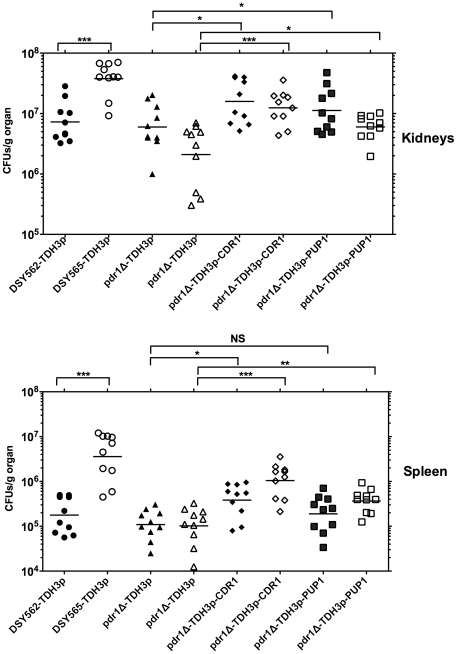
Effect of *CgCDR1* and *PUP1* overexpression on tissue colonization. **Panel A**: Fungal tissue burdens in kidneys. **Panel B**: Fungal tissue burdens in spleen. Tissue burden were determined from BALB/c mice infected intravenously with 4×10^7^ viable cells of *C. glabrata* strains. Mice were sacrificed at day 7 post-infection. Results are expressed as CFUs per gram of tissue and represent values recorded separately for each of the ten mice. Geometric means are indicated by horizontal bars and asterisks indicate statistically significant differences (*: *P*<0.05; **: *P*<0.01, ***: *P*<0.001). NS indicates no significance (*P*>0.05). Statistical differences were determined using the non-parametric Wilcoxon Rank sum tests (Prism 5.0). Strain background (DSY562 and DSY565) is indicated by filled or empty symbols, respectively. For strains derived from DSY562, the indicated names correspond to the following strains: DSY562-TDH3p: SFY196; *pdr1*Δ-TDH3p: SFY198; *pdr1*Δ-TDH3p-*CDR1*: SFY200 *pdr1*Δ-TDH3p-*PUP1*: SFY202. For strains derived from DSY565, the indicated names correspond to the following strains: DSY562-TDH3p: SFY197; *pdr1*Δ-TDH3p: SFY199; *pdr1*Δ-TDH3p-*CDR1*: SFY201 *pdr1*Δ-TDH3p-*PUP1*: SFY203.

The strains were next injected intravenously in mice and tissue burden were next assessed from kidneys and spleen from sacrificed animals (Fig. 8). In general, when *CgCDR1* and *PUP1* were overexpressed in a *pdr1*Δ mutant background, tissue burdens were significantly increased as compared to the parent strains. The colonization was slightly lower when *PUP1* was overexpressed as compared to *CgCDR1*.

**Figure 9:**
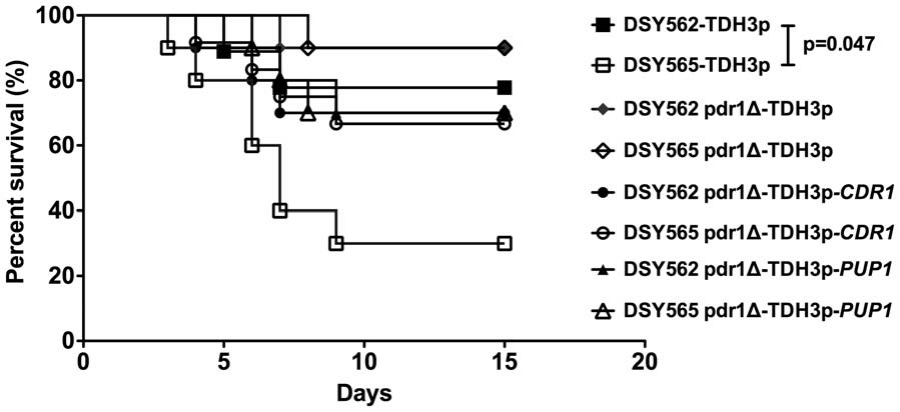
Virulence of *C. glabrata* in strains overexpressing *CgCDR1* and *PUP1*. Immuno-suppressed mice were infected as described in Material and Methods with strain derived from DSY562 and DSY565. Statistical differences were performed using the Log-rank Mantel-Cox test (Prism 5.0) by comparing survival curves of mice infected by the strains as indicated. The comparison between DSY565-TDH3p and DSY565-TDH3p was significant (p = 0.04) while comparisons of strains overexpression *CgCDR1* and *PUP1* with parents (*pdr1*Δ-TDH3) was not significant. See legend of Fig. 8 for strain designations.

When virulence of the same strains was tested in the immuno-suppressed mice model, the results showed no significant difference between strains overexpressing *CgCDR1* or *PUP1* as compared to the *pdr1*Δ mutants (Fig. 9). A closer inspection of the obtained data still suggests that strains overexpressing *CgCDR1* or *PUP1* tended to be more virulent than their parents. At day 15 post-infection, 90% of the mice infected with the *pdr1*Δ mutants survived, while approximately 70% survived when infected with the overexpressing strains (Fig. 9).

These results support the idea that the individual overexpression of *CgCDR1* and *PUP1* contributed moderately to virulence, however their overexpression was more important for maintaining tissue colonization. Taken together, our results indicate that both *CgCDR1* and *PUP1* are important mediators of *C. glabrata* virulence, but that their individual overexpression *per se* is not sufficient to mimic the increased virulence conferred by *CgPDR1* GOF mutations.

## Discussion

In this study we analysed the expression profiles of GOF mutations obtained from azole-resistant isolates in a previous study [13]. The analysis of transcription profiles gave only two genes commonly upregulated by all GOFs, *CgCDR1* and *PUP1*. Other investigators have analysed transcription profiles of azole-resistant isolates and thus enable comparisons with our study. Recently, Tsai *et al.* [16] obtained the transcription profiles of seven clinical pairs, each containing an azole-susceptible and an azole-resistant isolate. The *CgPDR1* GOF obtained from these strains were different from those investigated here, except for the L280F GOF. Their study highlighted 45 genes regulated (by ≥2-fold change as compared to the susceptible parent) by at least one clinical pair. Our study revealed a larger set of genes regulated by at least one GOF (i.e. 626 genes). *CgCDR1* and *PUP1*, the two genes selected in our study were found commonly upregulated by all GOFs in the Tsai *et al.* [16] study including by decreasing expression levels, *CgCDR1*, CAGL0M12947g (*PUP1*), CAGL0F02717g (*CgCDR2/PDH1*), CAGL0K00715g (*RTA1*), CAGL0C03289g (*YBT1*), CAGL0G00242g (*YOR1*), CAGL0K09702g, CAGL0A00451g (*CgPDR1*) and CAGL0G01122g. In a study published by Vermitsky *et al.* [14], one azole-resistant isolate (F15) was compared to an azole-susceptible parent. From the 109 genes regulated by at least two-fold in the resistant isolate, 34 were found regulated (out of 626 genes) in our study, among which *CgCDR1* and *PUP1*, the latter being the most upregulated gene in their study. The differences in transcriptional profiles could be explained by several factors including experimental conditions, type of array technology and intrinsic differenced between isolates used in all three studies. One major difference between our study and others is that we used an isogenic background in the reintroduction of the seven individual *CgPDR1* alleles, which prevents intrinsic strain variations. This is perhaps a reason for the difference between the number of genes regulated in at least one condition in our study (626 genes regulated by at least one GOF) and that of Tsai *et al.* [16] (45 genes regulated in at least one strain pair). This view is supported by separate results obtained with the transcriptional comparison of two related clinical strains, DSY717 and DSY2317, the latter containing the *CgPDR1* GOF L1081F. Between these two isolates, only 39 genes were regulated by at least two-fold (File S3), including *CgCDR1* and *PUP1*, thus suggesting that intrinsic strain variations may mask the real effect of GOF on the *C. glabrata* transcriptome.

The overlap between our study and others [14], [16] falls into 14 regulated genes (Figure S4). Besides *CgCDR1* and *PUP1*, which were found consistently upregulated in all three studies, the other genes may constitute a core set of genes regulated by *CgPDR1*. It is interesting to observe that the 14 genes are almost all found upregulated in the data provided by Vermitsky *et al.* [14] and Tsai *et al.* [16], while in our case, the regulation of these genes is dependent on the type of reintroduced GOF in the same genetic background. Several hypotheses will be provided below.

Given that *CgPDR1* is a major regulator of azole resistance in *C. glabrata* and should act on regulated genes via PDRE binding elements in the promoters of regulated genes, the consensus for *CgPDR1* binding (TCCRYGSR) was proposed and we searched systematically for this motif in the promoter regions of the 626 genes regulated by at least one GOF in our study. Fourty six (46) genes contained the consensus. We asked whether the degree of upregulation obtained by each GOF could be associated by the presence of the consensus. Our data show that the PDRE consensus was present in seven (for SFY101) to 45% (for SFY115) of the upregulated genes in single to several copies (see File S1). The presence of the PDRE could be detected in the downregulated genes, however the proportion was low (between 1–4%) and usually the detected PDRE occurred in a single copy. Regulatory elements on genes dependent on individual GOF were also searched with the RSAT tool (File S4). The following consensus site (TCCACGGA) could be detected in the promoters of upregulated genes from the GOF L280F (SFY115) and P822L (SFY116) and D1082G (SFY103). It resembles the PDRE consensus proposed by Vermitsky *et al.* [14] and fits to the sequence TCCACGGA published by Tsai *et al.* [16]. In complement to these analyses, we also observed that the PDRE consensus was present in 11 out of 14 promoters of regulated genes from three different data sets (Figure S4) and thus highlights the relevance of this binding site for the regulation of these genes. Future studies will be needed to address the genome-wide occupancy of CgPdr1 by chromatin immuno-precipitation experiments in *C. glabrata*. One can expect that CgPdr1 will bind to some extent to the genes commonly regulated by the different studies discussed here.

We showed here that GOF mutations in *CgPDR1* have differential effect on transcriptional profiles. This result was unexpected since previous results investigating the effect of GOF mutations in regulators of drug resistance in other yeast species (for example *MRR1* or *TAC1* mutations in *C. albicans*) have concluded to a convergence of transcriptional profiles with different mutations on a same regulator [22], . As mentioned from data shown in Fig. 1A, while a pairwise comparison between two GOFs can yield good correlation between expressed genes, another example between R376W and P822L gave striking different results: here, about 55% of the regulated genes showed an inverse expression pattern. Such patterns is not unique to our study: Tsai *et al.* [16] have analysed the expression of a few genes including *CgCDR1*, *CgPDR1*, *CgSNQ2* in a set of isogenic strains into which individual GOF were re-introduced. The authors observed a GOF-dependent gene expression pattern as documented here. Presently, no clear explanations could be provided four our observations. However, taking *S. cerevisiae* homologues Pdr1 and Pdr3 as models, some hypothesis can be formulated. In *S. cerevisiae*, the expression of the ABC-transporters *PDR5*, *SNQ2*, *PDR10*, *PDR15* and *YOR1* is controlled by Pdr1p/Pdr3p. In addition, Yrr1p modulates the expression of both *SNQ2* and *YOR1*. Similarly to *PDR3*, *YRR1* is autoregulated via PDREs in its promoter [25], [26]. Pdr1p and Pdr3p can act as homo- or heterodimers and can positively or negatively regulate expression of target genes, indicating that additional factors can modulate their activity [27], . For instance, the transcriptional regulator Rdr1p, acts as a repressor of *PDR5* in a PDRE-dependent manner and heterodimers of Rdr1p/Pdr1p or Rdr1p/Pdr3p compete with Pdr1p/Pdr3p for binding to PDREs [29], [30]. Similarly, the zinc cluster protein Stb5p also acts through PDREs and forms predominantly heterodimers with Pdr1p (no interaction with Pdr3p or Yrr1p yet described). Yrr1p is only present as a homodimer [31]. Pdr1 and Pdr3 can also associate to different subunits of the Mediator complex including Med15 and Med12, which is an important step into the recruitment of RNA polymerase II for target gene transcription. These two subunits are present in the C- and L-Mediator complexes, which may act as positive and negative regulator of transcription, respectively [32]. While both Pdr1 and Pdr3 can bind to Med15, Pdr3 binds in a specific manner to Med12 only in cells with mitochondrial dysfunctions [32]. With respect to CgPdr1, which combined in a single gene properties shared by Pdr1 and Pdr3, these studies suggest that CgPdr1 may interact with other DNA-binding proteins and may also associate with different subunits of the Mediator complex. The different GOF detected in CgPdr1 may alter in a positive or negative manner these interactions and thus could result in differentiated gene expression patterns as observed in our study. Future studies will be needed to verify this hypothesis.

Virulence and tissue burden quantitative assays performed in this study support the idea that *CgCDR1* and *PUP1* are important for the pathogenesis of *C. glabrata* at some stage of the infection. Currently our data cannot discriminate whether or not *C. glabrata* can replicate in the tested animal models. At least, the tested strains can persist over the time course of the experimentation, which is consistent with similar experiments performed in mice [33]. Interestingly, enhanced virulence has been observed in other *C. glabrata* isolates where azole resistance results from mitochondrial dysfunctions independently of GOF *CgPDR1* mutations. In this case, *CgCDR1* and *PUP1* are strongly upregulated and thus may also contribute to favor *C. glabrata* in host interactions [34]. The specific role of individual gene in fungal-host interaction remains to be solved however several reports have already identified ABC-transporters as able to contribute to selective advantages under host conditions. For example, the *Cryptococcus neoformans* ABC transporter *AFR1* was shown to interfere with lysosome acidification in macrophages to increase its survival. In particular, azole-resistant isolates showing increased *AFR1* expression were more virulent than their parental azole-susceptible isolates [35], [36], [37], which highlights the relevance of the association between drug resistance and virulence observed here. Interestingly, a recent study reported that *AFR1* upregulation could be obtained by reversible chromosome duplication and thus suggests *C. neoformans* could use this mechanism to modulate its virulence [38]. In another fungal species, *Botrytis cinerea*, which is a fungus causing losses of commercially important fruits, vegetables and vineyards worldwide, ABC-transporter upregulation was associated with drug resistance due to the use of fungicides. *B. cinerea* drug resistance is spreading, thus arguing against a fitness cost due to ABC-transporter upregulation [39]. Regarding *PUP1*, no other homologues were found yet involved in microbial pathogenesis and therefore the exact role of the product encoded by this gene in *C. glabrata* pathogenesis remains an open question.

We have attempted the overexpression of both genes in a *CgPDR1*-independent manner and animal experiments yielded results in favor of the hypothesis that *CgCDR1* and *PUP1* contribute to virulence. However, while tissue burden of mice were consistently increased when *CgCDR1* and *PUP1* were overexpressed (Fig. 8), virulence assays failed to discriminate in a statistical manner survival curves obtained with the overexpressing strains (Fig. 9). Several hypotheses could be provided explaining these results. First, it is possible that enhanced virulence needs the simultaneous overexpression of *CgCDR1* and *PUP1* to result in significant survival differences with parental strains. Second, it is also possible that, because the overexpression was carried out in a *pdr1*Δ mutant, other *CgPDR1*-dependent genes still need to be co-expressed for phenocopying the enhanced virulence of the original strain DSY565. Moreover, it is possible that the animal model used here (mouse intravenous infection) is not best suited to reveal the role of the two investigated genes. Urinary tract infection models might represent an alternative, as demonstrated by Domergue *et al.* [40]. These questions are currently being addressed in the laboratory.

In conclusion, our study started from a transcriptional analysis to identify important mediators of azole resistance and virulence in *C. glabrata*. The ABC transporter *CgCDR1* contributes almost solely to azole resistance but but has other activities contributing to the enhanced virulence of azole-resistant isolates. Nevertheless, this protein could be targeted for the design of inhibitors interfering both with resistance and virulence of this yeast species. ABC-transporter inhibitors have been already described and among them some are used in animal health for parasite protection (i.e. mylbemycins) and have low toxicity profiles for mammalian cells [41]. It will be therefore interesting to test these substances in the future to decrease drug resistance and its associated virulence in *C. glabrata*.

## Materials and Methods

### Strains and growth media

*C. glabrata* strains used in this study are listed in Table 4. Yeasts were grown in complete medium YEPD (1% Bacto peptone, Difco Laboratories, Basel, Switzerland), 0.5% Yeast extract (Difco) and 2% glucose (Fluka, Buchs, Switzerland). To prepare inocula for experimental infections, yeasts were grown in YEPD medium. When grown on solid media, 2% agar (Difco) was added. YPD agar plates containing nourseothricin (clonNAT, Werner BioAgents) at 200 mg ml^−1^ were used as a selective medium for growth of yeast transformant strains. *FLP*-mediated excision of the *SAT1* cassette was induced by growing the cells for 4 h at 30°C in YCB-BSA medium (23.4 g l^−1^ yeast carbon base and 4 g l^−1^ bovine serum albumin; pH 4.0). One hundred to 200 cells were then spread on YPD plates containing nourseothricin (15 µg ml^−1^) and grown for 48 h at 30°C to obtain nourseothricin-sensitive strains. This drug concentration can distinguish between nourseothricin-resistant and nourseothricin-sensitive cells. *Escherichia coli* DH5 was used as a host for plasmid construction and propagation. DH5α was grown in Luria-Bertani broth or on Luria-Bertani agar plates supplemented with ampicillin (0.1 mg ml^−1^) when required.

**Table 4:** Strains used in this study.

Strain	Parental strain	Genotype	Reference
DSY562	Related to DSY565	Azole-susceptible clinical strain	[11]
DSY565		Azole-resistant clinical strain	[11]
DSY717	Related to DSY2317	Azole-susceptible clinical strain	[13]
DSY2317		Azole-resistant clinical strain	[13]
SFY92	DSY562	*pdr1*Δ::*SAT1-FLIP*	[13]
SFY93	SFY92	*pdr1*Δ::*FRT*	[13]
SFY94	DSY565	*pdr1*Δ::*SAT1-FLIP*	[13]
SFY95	SFY94	*pdr1*Δ::*FRT*	[13]
SFY101	SFY93	*pdr1*Δ::*PDR1^R376W^-SAT1*	[13]
SFY103	SFY93	*pdr1*Δ::*PDR1^D1082G^-SAT1*	[13]
SFY105	SFY93	*pdr1*Δ::*PDR1^T588A^-SAT1*	[13]
SFY109	SFY93	*pdr1*Δ::*PDR1^E1083Q^-SAT1*	[13]
SFY111	SFY93	*pdr1*Δ::*PDR1^Y584C^-SAT1*	[13]
SFY114	SFY93	*pdr1*Δ::*PDR1-SAT1*	[13]
SFY115	SFY93	*pdr1*Δ::*PDR1^L280F^-SAT1*	[13]
SFY116	SFY93	*pdr1*Δ::*PDR1^P822L^-SAT1*	[13]
SFY148	DSY562	*cdr1*Δ::*SAT1-FLIP*	This study
SFY149	DSY565	*cdr1*Δ::*SAT1-FLIP*	This study
SFY150	DSY562	*pup1*Δ::*SAT1-FLIP*	This study
SFY151	DSY565	*pup1*Δ::*SAT1-FLIP*	This study
SFY152	SFY148	*cdr1*Δ::*FRT*	This study
SFY153	SFY149	*cdr1*Δ::*FRT*	This study
SFY154	SFY150	*pup1*Δ::*FRT*	This study
SFY155	SFY151	*pup1*Δ::*FRT*	This study
SFY159	SFY154	*pup1*Δ::*PUP1-SAT1*	This study
SFY160	SFY155	*pup1*Δ::*PUP1-SAT1*	This study
SFY161	SFY152	*cdr1*Δ::*CDR1-SAT1*	This study
SFY162	SFY153	*cdr1*Δ::*CDR1-SAT1*	This study
SFY167	DSY562	*CDR1_p_*::[pSF109]	This study
SFY168	DSY565	*CDR1_p_*::[pSF109]	This study
SFY169	SFY152	*cdr1*Δ::*FRT, pup1*Δ::*SAT1*	This study
SFY170	SFY153	*cdr1*Δ::*FRT, pup1*Δ::*SAT1*	This study
SFY173	DSY562	*PUP1*::[pSF113]	This study
SFY174	DSY565	*PUP1*::[pSF113]	This study
SFY196	DSY562	*ScTDH3_p_-SAT1*	This study
SFY197	DSY565	*ScTDH3_p_-SAT1*	This study
SFY198	SFY93	*pdr1*Δ::*FRT, ScTDH3_p_-SAT1*	This study
SFY199	SFY95	*pdr1*Δ::*FRT, ScTDH3_p_-SAT1*	This study
SFY200	SFY93	*pdr1*Δ::*FRT, ScTDH3_p_-CDR1-SAT1*	This study
SFY201	SFY95	*pdr1*Δ::*FRT, ScTDH3_p_-CDR1-SAT1*	This study
SFY202	SFY93	*pdr1*Δ::*FRT, ScTDH3_p_-PUP1-SAT1*	This study
SFY203	SFY95	*pdr1*Δ::*FRT, ScTDH3_p_-PUP1-SAT1*	This study

### Drug susceptibility assays

The *C. glabrata* strains were tested for azole susceptibility with the broth microdilution method described in the EUCAST document EDef 7.1 [42]. Briefly, aliquots of 1.5×10^5^ cells ml^−1^ were distributed into wells of a microtiter plate in RPMI 1640 containing 2% glucose and incubated at 35°C for 24 h. Endpoint readings were recorded with an automatic plate reader (Multiskan Ascent, Thermo) and the lowest azole concentration that reduced growth to 50% of that of the drug-free control was defined as the MIC.

### Construction of *C. glabrata* microarrays

The nucleotide sequences of the 5283 *C. glabrata* ORFs and the mitochondrial genome were downloaded from the Génolevure Consortium (http://www.genolevures.org/). Following the Agilent eArray Design guidelines, two separate probe sets for each ORF were designed, each consisting of 60 base oligonucleotides. The probe selection was performed using the GE Probe Design Tool. Probes were filtered following their base composition and distribution, cross-hybridization potential and melting temperature to yield two probe sets representing each 5210 nuclear and 6 mitochondrial ORFs. These probes cover more than 98% of the nuclear genome and represent 6 out of the 8 mitochondrial protein-encoding genes. For quality control and normalization purposes, 103 probes were selected randomly and spotted 20 times throughout each array in addition to standard Agilent controls including spike controls for intra- and inter-array normalizations. *C. glabrata* custom arrays were manufactured in the 8×15 k format by Agilent Technologies.

### cRNA synthesis, one-color labelling and *C. glabrata* arrays hybridization

Sample preparation was performed on three biological triplicates. Total RNA was extracted from log phase cultures in liquid YEPD as previously described [8]. Briefly, after centrifugation of 5 ml culture (corresponding to 10^8^ cells), the yeast cell pellet was mixed with 0.3 g of glass beads, 300 µl of RNA extraction buffer (0.1 M Tris-HCl at pH 7.5, 0.1 M LiCl, 10 mM EDTA, 0.5% SDS) and 300 µl of phenol-chloroform-isoamyl alcohol (24∶24∶1). After 1 min of vortexing in a bead beater (Fastprep-24 Instrument, MP Biomedicals Switzerland, Zürich), the aqueous phase was re-extracted with phenol-chloroform-isoamyl alcohol, and RNA was precipitated with 600 µl of ethanol at −20°C for 1 h. The RNA pellet was resuspended in 50 µl of diethyl pyrocarbonate-treated H_2_O. The integrity of the input template RNA has been determined prior to labeling/amplification, using Agilent RNA 6000 Nano LabChip kit and 2100 bioanalyzer (Agilent Technologies). Agilent's One-Color Quick Amp Labeling Kit (Agilent Technologies) was used to generate fluorescent cRNA according to the manufacturer's instructions. Briefly, 1 µg of total RNA from each sample was used to which a spike mix and T7 promoter primers were added, both of which are provided by the manufacturer. cDNA synthesis was promoted by MMLV-RT (Moloney Murine Leukemia Virus Reverse Transcriptase) in the presence of dNTPs and RNaseOUT. Next, cRNA was produced from this first reaction with T7 RNA polymerase, which simultaneously amplifies target material and incorporates cyanine 3-labeled CTP. The labelled cRNAs were purified with RNeasy Mini Kit (Qiagen) and quantified using NanoDrop ND-1000 UV-VIS Spectrophotometer. 600 ng of Cy3-labelled cRNAs were fragmented and hybridized for 17 h at 65°C to each array using the Gene Expression Hybridization Kit (Agilent Technologies) and a gasket slide with a 8 microarrays/slide format for sample hybridization to separate each sample in specific sub-arrays of the 8×15 K format.

### Microarrays data analysis

Slides were washed and processed according to the Agilent 60-mer Oligo Microarray Processing protocol and scanned on a Agilent microarray scanner G2565BA (Agilent Technologies). Data were extracted from the images with Feature Extraction (FE) software (Agilent Technologies). FE software flags outlier features, and detects and removes spatial gradients and local backgrounds. Data were normalized using a combined rank consistency filtering with LOWESS intensity normalization.

The gene expression values obtained from FE software were imported into GeneSpring 10.0.2 software (Agilent Technologies) for preprocessing and data analysis. For inter-array comparisons, a linear scaling of the data was performed using the 75th percentile signal value of all of non-control probes on the microarray to normalize one-color signal values. Probe sets with a signal intensity value below the 20th percentile were considered as absent and discarded from subsequent analysis. The expression of each gene was normalized by its median expression across all samples. Genes were included in the final data set if their expression changed by at least 2-fold between each strain expressing a *CgPDR1* GOF allele and the strain SFY114 expressing the *CgPDR1* wild type allele in at least two independent experiments. Corrected p-value (<0.05) was chosen as the cut-off for significance. Validation of genes found regulated by microarray analysis was performed by qRT-PCR analysis (see below for technical details) on a set of nine different genes. In general, the correlation found between qRT-PCR and microarray data was excellent (see Figure S1). Microarray data have been uploaded to the NCBI GEO microarray repository. The GEO accession number for the *C. glabrata* Agilent array is GPL10713 and the accession numbers for the data are GSE23827, GSE23828 and GSE23829.

### Use of bioinformatic tools

The analysis of consensus pattern on *C. glabrata* promoters (−800 to −1) was performed using the Regulatory Sequence Analysis Tools (RSAT: http://rsat.ulb.ac.be/rsat/index.html) and implemented to the pattern discovery tool (oligo-analysis). The settings were those supplied by default by the tool provider. The position-specific scoring matrices (PSSM) consensus matrices were converted using statistical parameters to consensus patterns and viewed via Weblogo [43].

GO term enrichment analysis in the investigated genes was carried out using the Generic Gene Ontology (GO) Term Finder online tool available at http://quantbio.princeton.edu/toolsResources.html.

### Quantitative real-time RT-PCR (qRT-PCR)

Total RNA was extracted from log phase cultures with an RNeasy Protect Mini kit (Qiagen) by a process involving mechanical disruption of the cells with glass beads and an RNase-free DNase treatment step as previously described [44]. Expression of the *CgCDR1*, *CgCDR2* and *CgSNQ2* genes was quantitatively assessed with real-time RT-PCR in an i-Cycler iQ system (Bio-Rad). All primers and probes [44] were designed with Beacon Designer 2 (version 2.06) software (Premier Biosoft International) and synthesized by MWG Biotech (Ebersberg, Germany). qRT-PCRwere carried out as previously described [44]. Each reaction was run in triplicate on three separate occasions. For relative quantification of the target genes, each set of primer pairs and the Taqman probes were used in combination with the primers and probe specific for the *CgACT1* reference gene in separate reactions [9].

*CgPDR1* and *PUP1* expression levels were determined by real-time qRT-PCR in a StepOne Real-time PCR System (Applied Biosystems) [13] using the Mesa Blue qPCR Mastermix Plus for Sybr assay kit (Eurogentec). Each reaction was run in triplicate on three separate occasions. *CgPDR1* and *PUP1* expression levels were normalized by *CgACT1* expression. Changes (*n*-fold) in gene expression relative to that of control isolate SFY114 were determined from *CgACT1-*normalized expression levels. The primers used for *PUP1* quantification are PUPa (5′-cactggtgcgctgaaaggtg-3′) and PUPb (5′-tgtcccaggctatctttgcc-3′). The primers used for *CgPDR1* and *CgACT1* quantification were previously described [13]. A two-fold increase in the expression level of each gene was arbitrarily considered as significant [9].

### Disruption and replacement of *CgCDR1*

For the disruption of *CgCDR1*, the *SAT1* flipping method was employed (Reuss *et al.*, 2004). The complete *CgCDR1* ORF flanked by 500 bp was amplified by PCR from genomic DNA of DSY562 using the primers CgCDR1-ApaI (5′-gcgcaaaGGGCCCtacatgttggcaaacccagg-3′) and CgCDR1-SacII (5′-gcgcaaaCCGCGGttggacaattgaatcagccg-3′) containing *Apa*I and *Sac*II restriction sites, respectively, and inserted into pBluescript II SK(+) to yield pSF87. *CgCDR1* deletion was created by PCR using the primers CgCDR1-XhoI (5′-gcgcaaaCTCGAGtgttacttttctttactttg-3′) and CgCDR1-NotI (5′-gcgcaaaGCGGCCGCtaatttatttagcctgcgct-3′) and pSF87 as a template. The resulting PCR product was digested with *Xho*I and *Not*I and ligated to a 4.7 kb *Xho*I-*Not*I fragment containing the *SAT1* flipper cassette from pSFS1 (referred as to *FLIP*) [45] to yield pSF91. This plasmid was linearised by digestion with *Apa*I and *Sac*II and transformed into DSY562 and DSY565. After selection of transformants on nourseothricin-containing YEPD plates (200 µg/ml), the *CgCDR1* deletion strains SFY148 and SFY149, respectively, were obtained.

For *CgCDR1* replacement, the *SAT1* cassette was excised in SFY148 and SFY149 to obtain the nourseothricin-sensitive strains SFY152 and 153 respectively. The 600-bp of the 3′UTR of *CgCDR1* ORF was amplified by PCR from DSY562 genomic DNA using the primers CgCDR1-NotIb (5′-gcgcaaaGCGGCCGCaaattttagacagcgctcgg-3′) and CgPDR1-SacIIb (5′-gcgcaaaCCGCGGtttgcgacaaattgggcagc-3′) and inserted into pSFS1 to yield pSF97. The complete *CgCDR1* ORF flanked by 500-bp upstream and 250-bp downstream was amplified using the primers CgCDR1-ApaI (see above) and CgCDR1-XhoIb (5′-gcgcaaaCTCGAGtatacctatgagcagatttc-3′) and inserted into pSF97 to yield pSF103. This plasmid was linearised by *Apa*I and *Sac*II and transformed into SFY152 and SFY153. After selection of transformants on, the *CgCDR1* revertant strains SFY161 and SFY162 were obtained.

### Disruption and replacement of *PUP1*

For the disruption of *PUP1* (CAGL0M12947g), the complete *PUP1* ORF flanked by 500-bp was amplified using the primers PUP-KpnI (5′-gcgcaaaGGTACCcattcatacccattccgtgg-3′) and PUP-SacI (5′-gcgcaaaGAGCTCtaggattcctgaaatgctgg-3′) containing *Kpn*I and *Sac*I restriction sites, and inserted into pBluescript II SK(+) to yield pSF90. *PUP1* deletion was created by PCR using the primers PUP-ApaI (5′-gcgcaaaGGGCCCattgtaacttatgttgtctg-3′) and PUP-SacII (5′-gcgcaaaCCGCGGagtgaccatactacacatta-3′) and pSF90 as a template. The resulting PCR product was digested with *Apa*I and *Sac*II and ligated to a 4.7 kb *Apa*I-*Sac*II fragment containing the *SAT1* flipper cassette from pSFS1 [45] to yield pSF94. This plasmid was linearised by digestion with *Kpn*I and *Sac*I and transformed into DSY562 and DSY565 to obtain the *PUP1* deletion strains SFY150 and SFY151, respectively.

Another *PUP1* deletion cassette was constructed to obtain strains with deletion in both *CgCDR1* and *PUP1*. As described above, pSF90 was amplified using the primers PUP-ApaI and PUP-SacII. The *SAT1* marker without the flipper system was amplified using the primers SAT1-ApaI (5′-gcaaaGGGCCCggaccacctttgattgtaaatagt-3′) and SAT1-SacII 5′-(ataagaatCCGCGGgtcaaaactagagaataataaag-3′) and pSFS1 as template. The resulting PCR products were digested with *Apa*I and *Sac*II and ligated to yield pSF101. This plasmid was transformed into the *CgCDR1* deletion strains SFY148 and SFY149 to obtain the *CgCDR1* and *PUP1* double deletion strains SFY169 and SFY170, respectively.

For *PUP1* replacement, the *SAT1* cassette was excised in SFY150 and SFY151 to obtain the nourseothricin-sensitive strains SFY154 and SFY155 respectively. *PUP1* replacement cassette was created by PCR using the primers PUP-ApaIb (5′-gcgcaaaGGGCCCcgaatctattggtcgcaagg-3′) and PUP-SacIIb (5′- gcgcaaaCCGCGGgtaagtcatggagcttatgc-3′) and pSF90 as a template. The resulting PCR product was digested with *Apa*I and *Sac*II and ligated to a 4.7 kb *Apa*I-*Sac*II fragment containing the *SAT1* flipper cassette from pSFS1 [45] to yield pSF98. This plasmid was linearised by *Kpn*I and *Sac*I and transformed into SFY154 and SFY155 to obtain the *PUP1* revertant strains SFY159 and SFY160. All above-constructed strains were verified by Southern blot analysis (see Figure S2). Transformants were selected onto nourseothricin-containing YEPD plates.

### Overexpression of *CgCDR1* and *PUP1*

For *CgCDR1* and *PUP1* overexpression, the *SAT1* marker was amplified using the primers SAT1-NotI (5′-ataagaatGCGGCCGCgtcaaaactagagaataataaag-3′) and SAT1-BamHI (5′-gcaaaGGATCCggaccacctttgattgtaaatagt-3′) and inserted into the *Not*I-*Bam*HI restriction sites of pBluescript II SK(+) to yield pSF30. This plasmid was then digested with *Xho*I and *Eco*RI and ligated to a 1.3 kb *Xho*I-*Eco*RI fragment containing the *C. glabrata CEN-ARS* from pCgACU-5 (Kitada *et al.*, 1996) to yield pSF126. The 0.7 kb *Eco*RI-*Bam*HI fragment from yEpGAP-Cherry-MCS [46] containing the constitutive *S. cerevisiae TDH3* promoter, was ligated into pSF126 to yield pSF127. The complete *CgCDR1* and *PUP1* ORFs were amplified by PCR from genomic DNA of DSY562 using the primers CgCDR1-EcoRIfor (5′-actGAATTCatgtctcttgcaagtgacaag-3′) and CgCDR1-EcoRIrev (5′-ataGAATTCtatacctatgagcagatttc-3′), and PUP-EcoRIfor (5′-actGAATTCatgtcagacagcagggaaat-3′) and PUP-EcoRIrev (5′-ataGAATTCcgaatctattggtcgcaagg-3′), respectively. The resulting PCR products were digested by *Eco*RI and inserted downstream of the *TDH3* promoter of pSF127 to yield the *CgCDR1* and *PUP1* overexpressing vectors, pSF129 and pSF130, respectively.

The plasmids pSF129 and pSF130 were transformed into the *PDR1* deletion strains SFY93 and SFY95 to obtain strains overexpressing *CgCDR1* (SFY200 and SFY201) or *PUP1*, (SFY202 and SFY203). As controls, plasmid pSF127 was introduced in strains DSY562, DSY565 and derivatives *pdr1*Δ mutants SFY93 and SFY95 to yield strains SFY196, SFY197, SFY198 and SFY 199, respectively. Transformants were selected onto nourseothricin-containing YEPD plates.

### Construction of the fusions *CgCDR1p-*3x*GFP* and *PUP1*-3x*GFP*

To express *GFP* under the control of the *CgCDR1* promoter, the *SAT1* marker was amplified using the primers SAT1-StuI (5′-ataagaatAGGCCTgtcaaaactagagaataataaag-3′) and SAT1-BamHI (see above) and inserted into the *Stu*I-*Bgl*II restriction sites of pBS-3xGFP–TRP1 [47] containing three tandemly fused *GFP* genes (3x*GFP*) to yield pSF104. Five hundred bp of the *CgCDR1* promoter were amplified from genomic DNA of using the primers CgCDR1p-BamHI (5′-gcgcaaaGGATCCtacatgttggcaaacccagg-3′) and CgCDR1p-BclI (5′-gcgcaaaTGATCAtgttacttttctttactttg-3) containing *Bam*HI and *Bcl*I restriction sites, respectively, and inserted into the *Bam*HI site of pSF104 to yield pSF109. This plasmid was linearised by digestion with *Sph*I and transformed into DSY562 and DSY565 to obtain strains SFY167 and SFY168, respectively.

To fuse the 3x*GFP* gene and the *PUP1* ORF, the complete *PUP1* ORF was amplified from DSY562 genomic DNA using the primers PUP-BglIIf (5′-gcgcaaaAGATCTatgtcagacagcagggaaat-3′) and PUP-BglIIr (5′-gcgcaaaAGATCTtgtatgatcattatcctt-3′) and inserted into the *Bam*HI site of pSF104 to yield pSF113. This plasmid was linearised by digestion with *Nco*I and transformed into DSY562 and DSY565 to obtain strains SFY173 and SFY174, respectively. Transformants were selected onto nourseothricin-containing YEPD plates.

### Confocal microscopy

To label mitochondria, log phase cultures of strain SFY174 were treated with 0.25 µM Mitotracker® Red CMXRos (Molecular Probes) for 30 min and washed with PBS. *C. glabrata* cells were fixed in 3.5% para-formaldehyde at 4°C for 5 min followed by 10 min at room temperature. Cells were then washed 3–5 min with phosphate-buffered saline (10 mM Na_2_HPO_4_, 2 mM KH_2_PO_4_, 140 mM NaCl, 3 mM KCl, pH 7.4). The remaining fixative was quenched with 100 mM Tris-HCl, pH 8.0. Fluorescence was analyzed with a confocal fluorescence microscope (Zeiss LSM 510 Meta, Jena, Germany).

### Flow cytometry

Groups of four female BALB/c mice (20 to 25 g; Charles-River) were injected into their lateral vein with saline suspensions containing 4×10^7^ colony-forming units (CFU) of the *C. glabrata* strains (each in a volume of 250 µl). After seven days, mice were sacrificed by CO_2_ inhalation, and kidneys were excised aseptically and homogenized in 10 ml sterile water. Kidneys homogenates were washed twice with FACS buffer (1×PBS pH 7.4, 5% FCS, 2 mM EDTA pH 8.0) and resuspended in 2 ml FACS buffer. Remaining tissue aggregates and cell clumps were eliminated by filtration through 50-µm cell strainers. A FACSCalibur® system (BD Bioscience) and the CellQuest™ software were used for analysis.

### Animal studies

Female BALB/c mice (20 to 25 g) were purchased from Harlan Italy S.r.l (San Pietro al Natisone, Udine, Italy) and inbred in-house. The mice were housed in filter-top cages with free access to food and water. To establish *C. glabrata* infection, mice were injected into their lateral vein with saline suspensions of the *C. glabrata* strains (each in a volume of 200 µl).

In virulence studies, a group of ten immuno-suppressed mice was established for each yeast strain. Mice were rendered neutropenic by intraperitoneal administration of cyclophosphamide (200 mg kg^−^1 of body weight per day) three days before challenge and on the day of infection. Mice were injected with 7×10^7^ colony-forming units (CFU) of each of the investigated strains. For tissue burden experiments, immuno-competent mice were inoculated with 4×10^7^ CFU. After seven days, mice were sacrificed by use of CO_2_ inhalation, and target organs (spleen and kidney) were excised aseptically, weighted individually, and homogenized in sterile saline by using a Stomacher 80 device (Pbi International, Milan, Italy) for 120 s at high speed. Organ homogenates were diluted and plated onto YPD. Colonies were counted after two days of incubation at 30°C, and the numbers of CFU g^−1^ of organ were calculated. For survival experiments, mice were made neutropenic as previously described [48] and then injected with 7×10^7^ CFUs of each of the strains studied. Mice were monitored with twice-daily inspections and those that appeared moribund or in pain were sacrificed by use of CO_2_ inhalation.

CFU counts were analyzed with non-parametric Wilcoxon Rank sum tests, while mean survival times were compared among groups by using the long-rank test. A *P*-value of less than 0.05 was considered to be significant.

### Ethics Statement

The animal experiments were performed under a protocol approved by the Institutional Animal Use and Care Committee at Università Cattolica del S. Cuore, Rome, Italy (Permit number: L21, 10/02/2008) and authorized by the Italian Ministry of Health, according to Legislative Decree 116/92, which implemented the European Directive 86/609/EEC on laboratory animal protection in Italy. Animal welfare was routinely checked by veterinarians of the Service for Animal Welfare.

Animal experiments carried out for *in vivo* detection of GFP-tagged proteins (see above) were performed at the University of Lausanne and University Hospital Center under the surveillance of the local governmental veterinarian offices. These experiments were approved by the local governmental veterinarian offices and are registered under number 1734.2.

## Supporting Information

Figure S1 **Validation of microarrays results by qRT-PCR.** **Panel A**: Gene expression relative to the strain SFY114 (containing the wild type *CgPDR1* allele) obtained by microarray analysis for each of the investigated GOF mutation in *CgPDR1*. Color code for up- and downregulated genes is given. **Panel B**: Gene expression relative to the strain SFY114 obtained by qRT-PCR. The values are averages of three separate experiments and represent increase in gene expression relative to SFY114 (set at 1.00). Primers used for *CgPDR1*, *PUP1*, *CgCDR1* and the normalization control *CgACT1* are described in the Material and Methods section. Other primers used for qRT-PCR are listed below. The comparison between qRT-PCR results and microarrays was estimated by linear regression between relative expression changes. R^2^ values ranged from 0.4 and 0.89 between comparisons. Two comparisons including values obtained for CAGL0A00473g and CAGL0A00451g (*PDR1*) gave low correlation coefficients. This is explained by the fact that microarrays values of regulated genes were 10–100 fold different than observed for qRT-PCR. However, these discrepancies do not change the categorization of these genes being up- and downregulated by a given GOF mutation and taking a 2-fold change as a cut-off value. Forward and reverse primers are the following for CAGL0K00715g: 5′-TGCATCATCGAAGTCGTTGG-3′ and 5′-CCCACGAGTAACAGCACCACT-3′; for CAGL0E03894g: 5′-AAGCCGCAGACAAAGAGCAG-3′and 5′-CATCACCATTCTCGCCGTG-3′; for CAGL0A00473g: 5′-CACTGGTGCGCTGAAAGGTG-3′ and 5′-TGTCCCAGGCTATCTTTGCC-3′; for CAGL0F01111g: 5′-GTTTGGCTACTTGAGCACCGA-3′ and 5′-CGATCTCCCCTAGGCCATC-3′; for CAGL0I09724g: 5′-GCCTGAGAGCTTGGACCACT-3′ and 5′-TTGTTGGACGTGGTCTTCGA-3′; for CAGL0D02662g: 5′-CGCTGATGTTTCTGCGATGT-3′ and 5′-CACCGAATGCGATCATCAAA-3′.
(TIF)

Figure S2 **Southern blot analysis and diagram illustrating strategies for disruption and replacement of** ***CgCDR1*** **and** ***PUP1*** **in** ***C. glabrata*** **isolates.** DNA was purified from isolated colonies, digested with the restriction enzyme *Pvu*II, analyzed by gel electrophoresis and hybridized to specific probes. **Panel A**: Analysis of *CgCDR1* loci. The expected sizes for *CgCDR1* analysis are: 1.7 kb for DSY562 and DSY565 (wild type *CgCDR1* locus); 6.1 kb for SFY148 and SFY149 (*cdr1*Δ::*SAT1-*FLIP); 1.3 kb for SFY152, SFY153, SFY169 and SFY170 (*cdr1*Δ::*FRT*); 1.7 kb for SFY161 and SFY162 (*cdr1Δ*::*CgCDR1-SAT1*). **Panel B**: Analysis of *PUP1* loci. The expected sizes for *PUP1* analysis are: 1.2 kb for DSY562 and DSY565 (wild type *PUP1* locus); 12.6 kb for SFY150 and SFY151 (*pup1*Δ::*SAT1-*FLIP); 7.8 kb for SFY154 and SFY155 (*pup1*Δ::*FRT*); 1.2 kb for SFY159 and SFY160 (*pup1*Δ::*PUP1*-*SAT1*); 9.7 kb for SFY169 and SFY170 (*pup1*Δ::*SAT1*).
(TIF)

Figure S3 **Promoter consensus analysis of genes upregulated in SFY103 (GOF mutation D1082G) and SFY116 (GOF mutation P822L).** The data was obtained using RSAT (http://rsat.ulb.ac.be/rsat/index.html) and the oligo-analysis tool with default settings.
(TIF)

Figure S4 **Comparisons of transcript profiling experiments of azole resistance in** ***C. glabrata*** **.** **Panel A**: Venn diagram was obtained by comparisons of published studies [14], [16] with the present study and included all genes regulated by ≥2-fold. **Panel B**: List of the 14 genes commonly regulated as reported by published studies [14], [16] and by the present study. Color codes and abbreviations are detailed in File S1.
(TIF)

File S1 **List of genes regulated by the GOF mutations in** ***CgPDR1*** **.**
(XLSX)

File S2 **List of genes regulated by** ***CgPDR1*** **in** ***C. glabrata*** **.**
(XLSX)

File S3 **List of genes regulated in a pair of isolate containing an azole-susceptible (DSY717) and an azole-resistant isolate (DSY2317).**
(XLS)

File S4 **Putative regulatory sequences in genes regulated by GOF mutations in** ***CgPDR1*** **.**
(PDF)
