# Supplementary figures and images for: Contribution of CgPDR1-Regulated Genes in Enhanced Virulence of Azole-Resistant Candida glabrata
Source: PLoS One. 2011 Mar 9;6(3):e17589. doi: 10.1371/journal.pone.0017589 (PMC3052359; doi:10.1371/journal.pone.0017589)

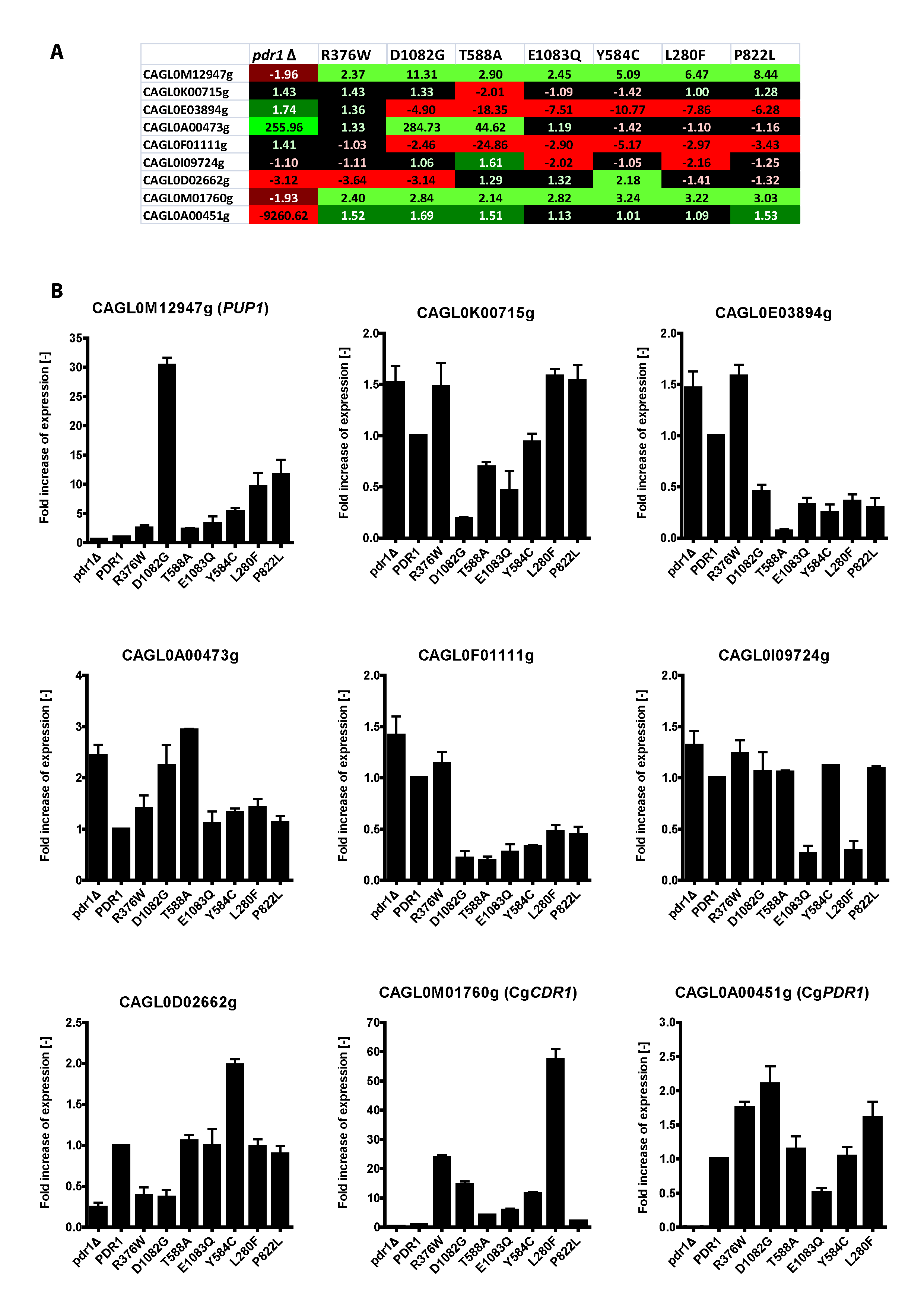

Supplement: Figure S1 — Validation of microarrays results by qRT-PCR. Panel A: Gene expression relative to the strain SFY114 (containing the wild type CgPDR1 allele) obtained by microarray analysis for each of the investigated GOF mutation in CgPDR1. Color code for up- and downregulated genes is given. Panel B: Gene expression relative to the strain SFY114 obtained by qRT-PCR. The values are averages of three separate experiments and represent increase in gene expression relative to SFY114 (set at 1.00). Primers used for CgPDR1, PUP1, CgCDR1 and the normalization control CgACT1 are described in the Material and Methods section. Other primers used for qRT-PCR are listed below. The comparison between qRT-PCR results and microarrays was estimated by linear regression between relative expression changes. R2 values ranged from 0.4 and 0.89 between comparisons. Two comparisons including values obtained for CAGL0A00473g and CAGL0A00451g (PDR1) gave low correlation coefficients. This is explained by the fact that microarrays values of regulated genes were 10–100 fold different than observed for qRT-PCR. However, these discrepancies do not change the categorization of these genes being up- and downregulated by a given GOF mutation and taking a 2-fold change as a cut-off value. Forward and reverse primers are the following for CAGL0K00715g: 5′-TGCATCATCGAAGTCGTTGG-3′ and 5′-CCCACGAGTAACAGCACCACT-3′; for CAGL0E03894g: 5′-AAGCCGCAGACAAAGAGCAG-3′and 5′-CATCACCATTCTCGCCGTG-3′; for CAGL0A00473g: 5′-CACTGGTGCGCTGAAAGGTG-3′ and 5′-TGTCCCAGGCTATCTTTGCC-3′; for CAGL0F01111g: 5′-GTTTGGCTACTTGAGCACCGA-3′ and 5′-CGATCTCCCCTAGGCCATC-3′; for CAGL0I09724g: 5′-GCCTGAGAGCTTGGACCACT-3′ and 5′-TTGTTGGACGTGGTCTTCGA-3′; for CAGL0D02662g: 5′-CGCTGATGTTTCTGCGATGT-3′ and 5′-CACCGAATGCGATCATCAAA-3′. (TIF) [file pone.0017589.s001.tif]

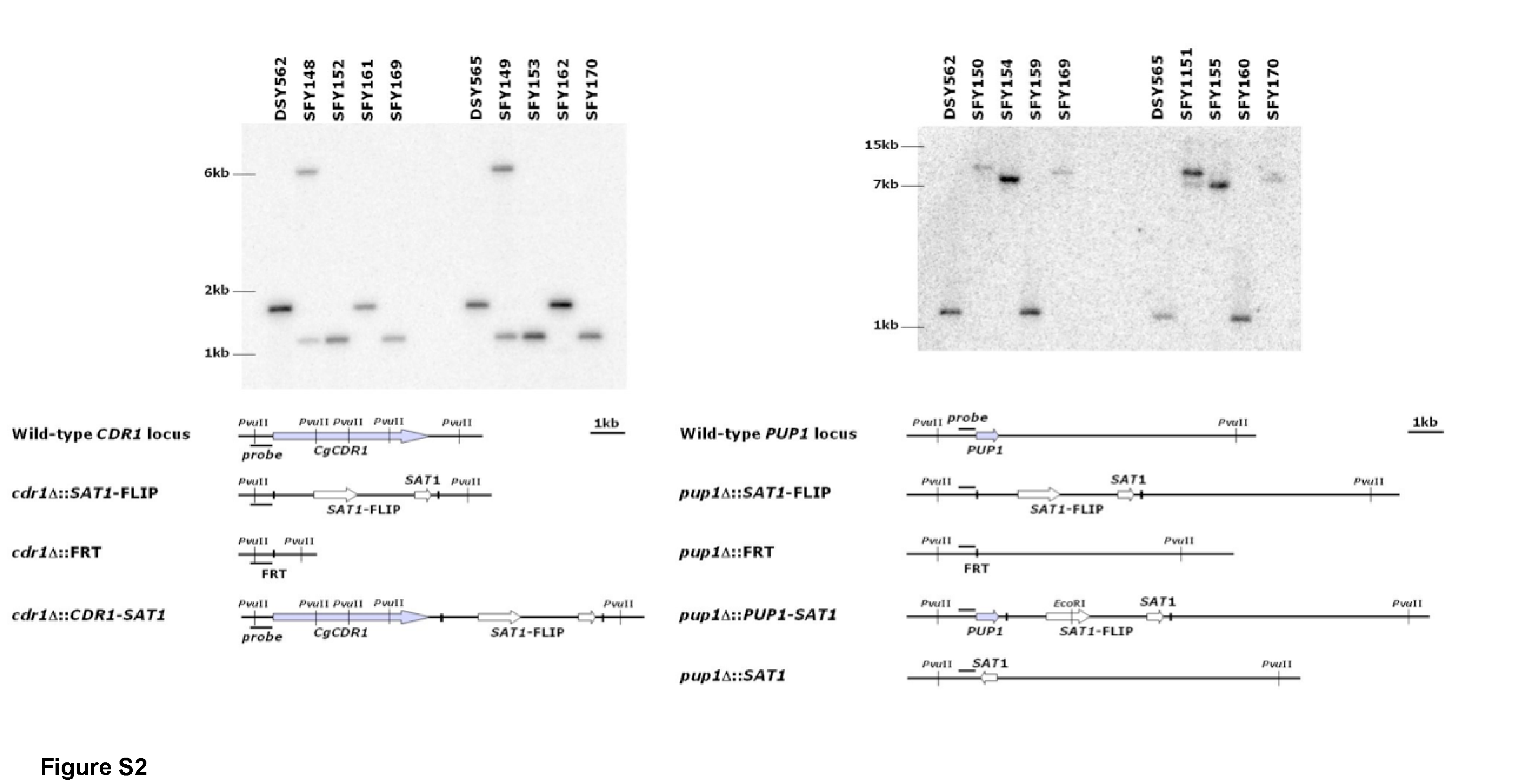

Supplement: Figure S2 — Southern blot analysis and diagram illustrating strategies for disruption and replacement of CgCDR1 and PUP1 in C. glabrata isolates. DNA was purified from isolated colonies, digested with the restriction enzyme PvuII, analyzed by gel electrophoresis and hybridized to specific probes. Panel A: Analysis of CgCDR1 loci. The expected sizes for CgCDR1 analysis are: 1.7 kb for DSY562 and DSY565 (wild type CgCDR1 locus); 6.1 kb for SFY148 and SFY149 (cdr1Δ::SAT1-FLIP); 1.3 kb for SFY152, SFY153, SFY169 and SFY170 (cdr1Δ::FRT); 1.7 kb for SFY161 and SFY162 (cdr1Δ::CgCDR1-SAT1). Panel B: Analysis of PUP1 loci. The expected sizes for PUP1 analysis are: 1.2 kb for DSY562 and DSY565 (wild type PUP1 locus); 12.6 kb for SFY150 and SFY151 (pup1Δ::SAT1-FLIP); 7.8 kb for SFY154 and SFY155 (pup1Δ::FRT); 1.2 kb for SFY159 and SFY160 (pup1Δ::PUP1-SAT1); 9.7 kb for SFY169 and SFY170 (pup1Δ::SAT1). (TIF) [file pone.0017589.s002.tif]

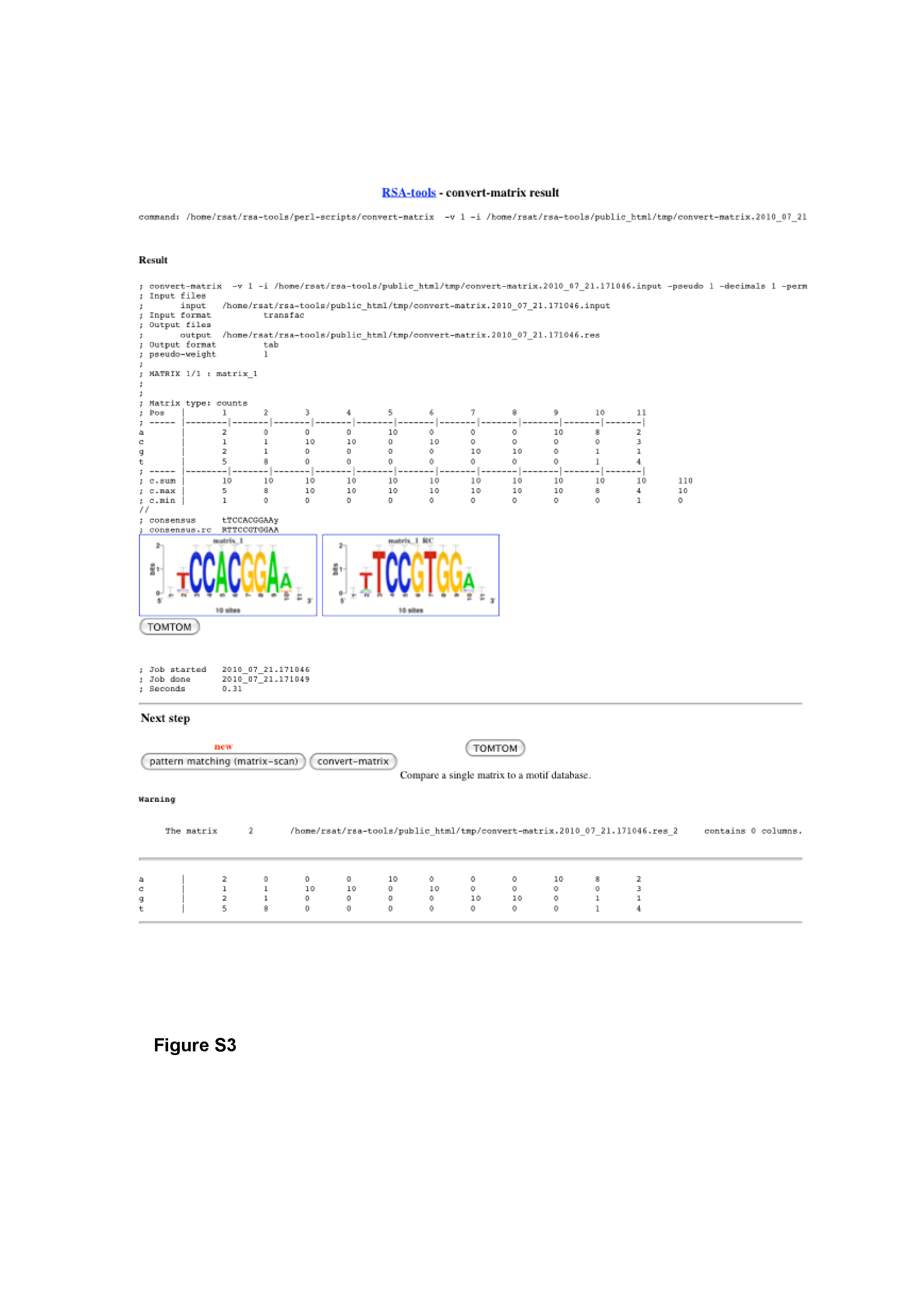

Supplement: Figure S3 — Promoter consensus analysis of genes upregulated in SFY103 (GOF mutation D1082G) and SFY116 (GOF mutation P822L). The data was obtained using RSAT (http://rsat.ulb.ac.be/rsat/index.html) and the oligo-analysis tool with default settings. (TIF) [file pone.0017589.s003.tif]

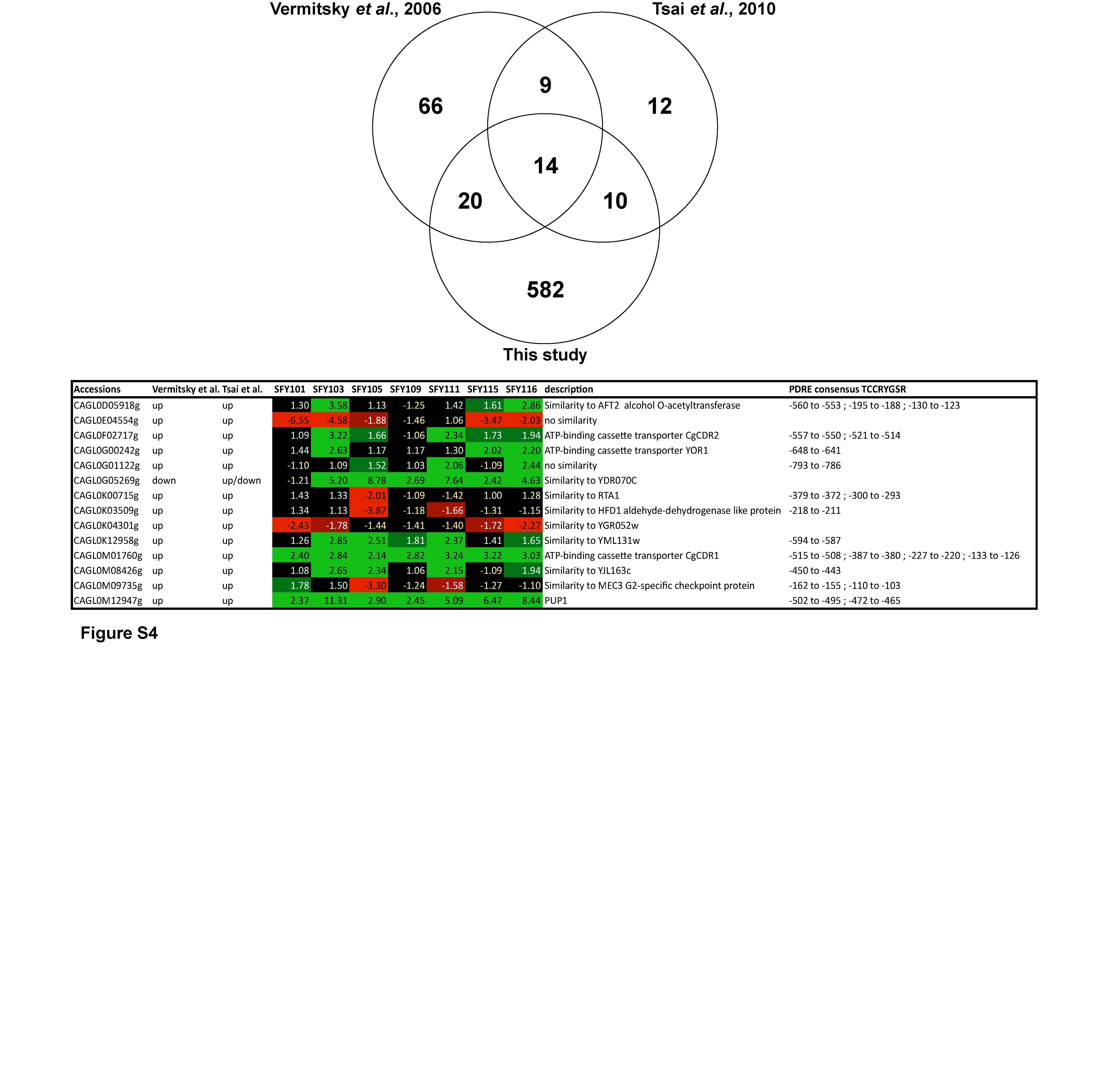

Supplement: Figure S4 — Comparisons of transcript profiling experiments of azole resistance in C. glabrata . Panel A: Venn diagram was obtained by comparisons of published studies [14], [16] with the present study and included all genes regulated by ≥2-fold. Panel B: List of the 14 genes commonly regulated as reported by published studies [14], [16] and by the present study. Color codes and abbreviations are detailed in File S1. (TIF) [file pone.0017589.s004.tif]
