# Supplementary material for: Contribution of CgPDR1-Regulated Genes in Enhanced Virulence of Azole-Resistant Candida glabrata
Source: PLoS One. 2011 Mar 9;6(3):e17589. doi: 10.1371/journal.pone.0017589 (PMC3052359; doi:10.1371/journal.pone.0017589)
Supplement: File S4 — Putative regulatory sequences in genes regulated by GOF mutations in CgPDR1 . (PDF) [file pone.0017589.s008.pdf]

RSAT-SFY103

RSA-tools - convert-matrix result

command: /home/rsat/rsa-tools/perl-scripts/convert-matrix -v 1 -i /home/rsat/rsa-tools/public_html/tmp/convert-matrix.2010_07_23

Result

```
; convert-matrix  -v 1 -i /home/rsat/rsa-tools/public_html/tmp/convert-matrix.2010_07_23.094031.input -pseudo 1 -decimals 1 -perm
; Input files
;       input   /home/rsat/rsa-tools/public_html/tmp/convert-matrix.2010_07_23.094031.input
; Input format          transfac
; Output files
;       output  /home/rsat/rsa-tools/public_html/tmp/convert-matrix.2010_07_23.094031.res
; Output format         tab
; pseudo-weight         1
;
; MATRIX 1/3 : matrix_1
;
;
; Matrix type: counts
; Pos   |       1       2       3       4       5       6       7       8       9       10      11      12
; ----- |-------|-------|-------|-------|-------|-------|-------|-------|-------|-------|-------|-------|
a       |       13      12      5       0       0       0       0       24      30      30      9       6
c       |       5       6       5       30      30      30      0       0       0       0       3       5
g       |       5       4       6       0       0       0       0       0       0       0       6       4
t       |       7       8       14      0       0       0       30      6       0       0       12      15
; ----- |-------|-------|-------|-------|-------|-------|-------|-------|-------|-------|-------|-------|
; c.sum |       30      30      30      30      30      30      30      30      30      30      30      30      360
; c.max |       13      12      14      30      30      30      30      24      30      30      12      15      30
; c.min |       5       4       5       0       0       0       0       0       0       0       3       4       0
//
; consensus     awtCCCTAAAwt
; consensus.rc  AWTTTAGGGAWT
```

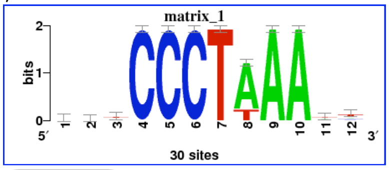

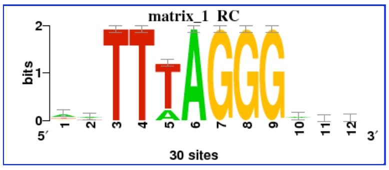

TOMTOM

```
;
; MATRIX 2/3 : matrix_2
;
;
; Matrix type: counts
; Pos   |       1       2       3       4       5       6       7       8       9       10      11      12      13
; ----- |-------|-------|-------|-------|-------|-------|-------|-------|-------|-------|-------|-------|-------|
a       |       7       3       1       0       0       14      0       0       0       14      13      3       4
c       |       2       0       0       14      14      0       14      0       0       0       0       2       4
g       |       3       2       1       0       0       0       0       14      14      0       1       2       5
t       |       2       9       12      0       0       0       0       0       0       0       0       7       1
; ----- |-------|-------|-------|-------|-------|-------|-------|-------|-------|-------|-------|-------|-------|
; c.sum |       14      14      14      14      14      14      14      14      14      14      14      14      14      182
; c.max |       7       9       12      14      14      14      14      14      14      14      13      7       5       14
; c.min |       2       0       0       0       0       0       0       0       0       0       0       2       1       0
//
; consensus     atTCCACGGAAtv
; consensus.rc  BATTCCGTGGAAT
```

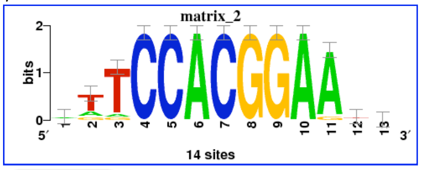

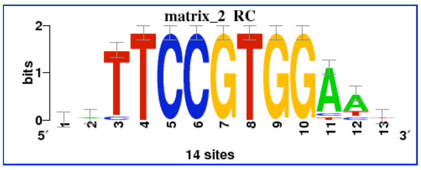

TOMTOM

```
;
; MATRIX 3/3 : matrix_4
;
;
; Matrix type: counts
; Pos   |       1       2       3       4       5       6       7       8       9       10      11
; ----- |-------|-------|-------|-------|-------|-------|-------|-------|-------|-------|-------|
a       |       8       6       0       0       18      0       0       0       0       6       2
c       |       4       2       0       0       0       0       0       0       0       1       5
g       |       4       0       0       0       0       18      18      18      6       3       4
t       |       2       10      18      18      0       0       0       0       12      8       7
; ----- |-------|-------|-------|-------|-------|-------|-------|-------|-------|-------|-------|
; c.sum |       18      18      18      18      18      18      18      18      18      18      18      198
; c.max |       8       10      18      18      18      18      18      18      12      8       7       18
; c.min |       2       0       0       0       0       0       0       0       0       1       2       0
//
; consensus     awTTAGGGkwy
; consensus.rc  RWMCCCTAAWT
```

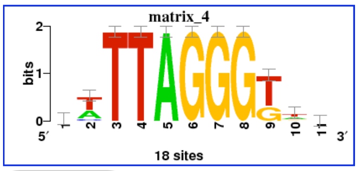

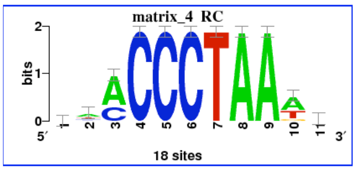

TOMTOM

```
; Job started   2010_07_23.094031
; Job done      2010_07_23.094037
; Seconds       0.31
```

Next step

new

pattern matching (matrix-scan) convert-matrix

TOMTOM

Compare a single matrix to a motif database.

Warning

The matrix 2 /home/rsat/rsa-tools/public_html/tmp/convert-matrix.2010_07_23.094031.res_2 contains 0 columns.

Warning

The matrix 4 /home/rsat/rsa-tools/public_html/tmp/convert-matrix.2010_07_23.094031.res_4 contains 0 columns.

Warning

The matrix 6 /home/rsat/rsa-tools/public_html/tmp/convert-matrix.2010_07_23.094031.res_6 contains 0 columns.

---

a	|	13	12	5	0	0	0	0	24	30	30	9	6
c	|	5	6	5	30	30	30	0	0	0	0	3	5
g	|	5	4	6	0	0	0	0	0	0	0	6	4
t	|	7	8	14	0	0	0	30	6	0	0	12	15

RSAT-SFY105

RSA-tools - convert-matrix result

```
command: /home/rsat/rsa-tools/perl-scripts/convert-matrix  -v 1 -i /home/rsat/rsa-tools/public_html/tmp/convert-matrix.2010
```

Result

```
; convert-matrix  -v 1 -i /home/rsat/rsa-tools/public_html/tmp/convert-matrix.2010_07_23.095136.input -pseudo 1 -decimals 1
; Input files
;       input   /home/rsat/rsa-tools/public_html/tmp/convert-matrix.2010_07_23.095136.input
; Input format          transfac
; Output files
;       output  /home/rsat/rsa-tools/public_html/tmp/convert-matrix.2010_07_23.095136.res
; Output format         tab
; pseudo-weight         1
;
; MATRIX 1/4 : matrix_1
;
;
; Matrix type: counts
; Pos   |       1       2       3       4       5       6       7       8       9       10      11      12      13      14
; ----- |-------|-------|-------|-------|-------|-------|-------|-------|-------|-------|-------|-------|-------|-------|
a       |       11      21      3       39      18      0       0       0       0       16      49      39      30      21
c       |       21      16      49      2       15      56      56      56      6       0       0       6       7       11
g       |       12      9       2       2       1       0       0       0       0       0       4       2       8       12
t       |       12      10      2       13      22      0       0       0       50      40      3       9       11      12
; ----- |-------|-------|-------|-------|-------|-------|-------|-------|-------|-------|-------|-------|-------|-------|
; c.sum |       56      56      56      56      56      56      56      56      56      56      56      56      56      56
; c.max |       21      21      49      39      22      56      56      56      50      40      49      39      30      21
; c.min |       11      9       2       2       1       0       0       0       0       0       0       2       7       11
//
; consensus     cmCAhCCCTWAAaa
; consensus.rc  TTTTWAGGGDTGKG
```

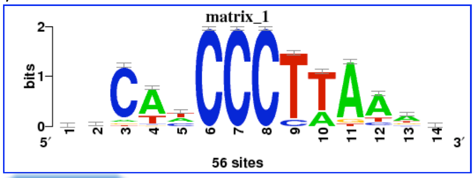

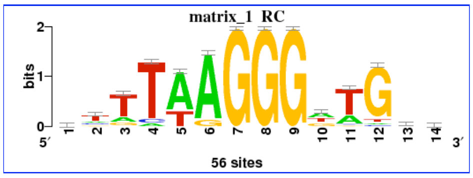

TOMTOM

```
;
; MATRIX 2/4 : matrix_2
;
;
; Matrix type: counts
; Pos   |       1       2       3       4       5       6       7       8       9       10      11      12      13
; ----- |-------|-------|-------|-------|-------|-------|-------|-------|-------|-------|-------|-------|-------|
a       |       14      10      0       0       42      0       0       0       42      8       4       11      7
c       |       7       8       0       0       0       21      0       42      0       7       8       6       6
g       |       8       9       0       42      0       21      0       0       0       4       4       12      12
t       |       13      15      42      0       0       0       42      0       0       23      26      13      17
; ----- |-------|-------|-------|-------|-------|-------|-------|-------|-------|-------|-------|-------|-------|
; c.sum |       42      42      42      42      42      42      42      42      42      42      42      42      42      546
; c.max |       14      15      42      42      42      21      42      42      42      23      26      13      17      42
; c.min |       7       8       0       0       0       0       0       0       0       4       4       6       6       0
//
; consensus     wtTGAsTCAttdk
; consensus.rc  MHAATGASTCAAW
```

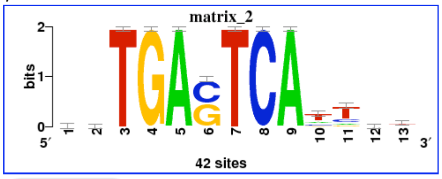

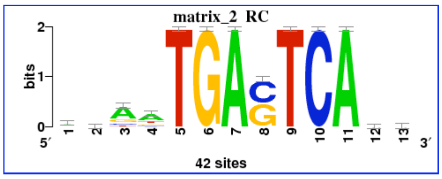

TOMTOM

```
;
; MATRIX 3/4 : matrix_4
;
;
; Matrix type: counts
; Pos   |       1       2       3       4       5       6       7       8       9       10      11      12      13      14
; ----- |-------|-------|-------|-------|-------|-------|-------|-------|-------|-------|-------|-------|-------|-------|
a       |       24      26      8       1       30      63      27      0       0       5       63      17      19      18
c       |       6       4       0       1       0       0       0       0       0       0       0       21      14      15
g       |       8       2       1       1       0       0       36      63      63      40      0       16      10      15
t       |       25      31      54      60      33      0       0       0       0       18      0       9       20      15
; ----- |-------|-------|-------|-------|-------|-------|-------|-------|-------|-------|-------|-------|-------|-------|
; c.sum |       63      63      63      63      63      63      63      63      63      63      63      63      63      63
; c.max |       25      31      54      60      33      63      36      63      63      40      63      21      20      18
; c.min |       6       2       0       1       0       0       0       0       0       0       0       9       10      15
//
; consensus     wwTTwArGGkAvwa
```

; consensus.rc   TWBTMCCYTWAAWW

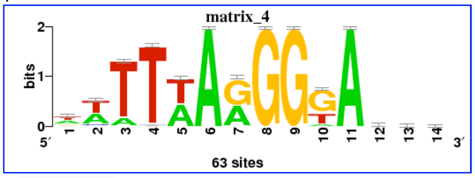

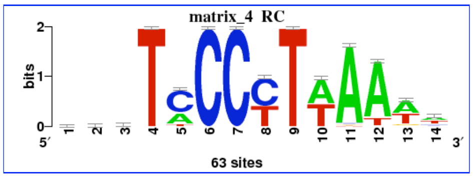

TOMTOM

;
; MATRIX 4/4 : matrix_5
;
;
; Matrix type: counts

; Pos	1	2	3	4	5	6	7	8	9	10	11	12	13	14
a	13	15	11	4	38	55	0	0	0	20	7	25	15	15
c	12	13	0	10	0	0	0	0	0	0	1	11	9	15
g	6	5	1	0	0	1	56	56	56	8	0	10	11	10
t	25	23	44	42	18	0	0	0	0	28	48	10	21	16
; c.sum	56	56	56	56	56	56	56	56	56	56	56	56	56	56
; c.max	25	23	44	42	38	55	56	56	56	28	48	25	21	16
; c.min	6	5	0	0	0	0	0	0	0	0	0	10	9	10

//
; consensus   twTTwAGGGwTawh
; consensus.rc   DWTAWCCCTWAAWA

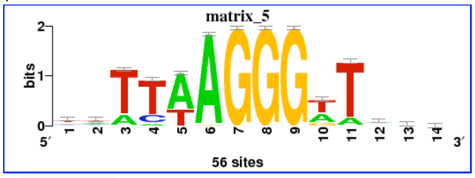

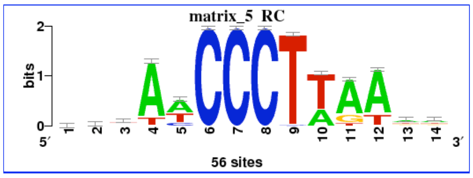

TOMTOM

; Job started   2010_07_23.095137
; Job done   2010_07_23.095144
; Seconds   0.35

Next step

new

pattern matching (matrix-scan)   convert-matrix   TOMTOM

Compare a single matrix to a motif database.

Warning

The matrix   2   /home/rsat/rsa-tools/public_html/tmp/convert-matrix.2010_07_23.095136.res_2   contains 0 col

---

Warning

The matrix   4   /home/rsat/rsa-tools/public_html/tmp/convert-matrix.2010_07_23.095136.res_4   contains 0 col

---

Warning

The matrix   6   /home/rsat/rsa-tools/public_html/tmp/convert-matrix.2010_07_23.095136.res_6   contains 0 col

---

Warning

The matrix   8   /home/rsat/rsa-tools/public_html/tmp/convert-matrix.2010_07_23.095136.res_8   contains 0 col

---

a	11	21	3	39	18	0	0	0	0	16	49	39	30	21
c	21	16	49	2	15	56	56	56	6	0	0	6	7	11
g	12	9	2	2	1	0	0	0	0	0	4	2	8	12

t	12	10	2	13	22	0	0	0	50	40	3	9	11	12

RSAT-SFY109

RSA-tools - convert-matrix result

```
command: /home/rsat/rsa-tools/perl-scripts/convert-matrix  -v 1 -i /home/rsat/rsa-tools/public_html/tmp/convert-matrix.2010_07_23
```

Result

```
; convert-matrix  -v 1 -i /home/rsat/rsa-tools/public_html/tmp/convert-matrix.2010_07_23.100039.input -pseudo 1 -decimals 1 -perm
; Input files
;       input   /home/rsat/rsa-tools/public_html/tmp/convert-matrix.2010_07_23.100039.input
; Input format          transfac
; Output files
;       output  /home/rsat/rsa-tools/public_html/tmp/convert-matrix.2010_07_23.100039.res
; Output format         tab
; pseudo-weight         1
;
; MATRIX 1/2 : matrix_1
;
;
; Matrix type: counts
; Pos   |       1       2       3       4       5       6       7       8       9       10      11      12
; ----- |-------|-------|-------|-------|-------|-------|-------|-------|-------|-------|-------|-------|
a       |       4       4       7       0       0       0       14      0       0       0       4       3
c       |       5       4       1       0       14      0       0       0       0       14      2       3
g       |       1       3       4       14      0       0       0       14      14      0       2       7
t       |       4       3       2       0       0       14      0       0       0       0       6       1
; ----- |-------|-------|-------|-------|-------|-------|-------|-------|-------|-------|-------|-------|
; c.sum |       14      14      14      14      14      14      14      14      14      14      14      14      168
; c.max |       5       4       7       14      14      14      14      14      14      14      6       7       14
; c.min |       1       3       1       0       0       0       0       0       0       0       2       1       0
//
; consensus     hmrGCTAGGCwg
; consensus.rc  CWGCCTAGCYKD
```

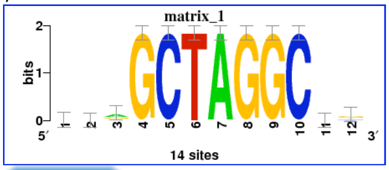

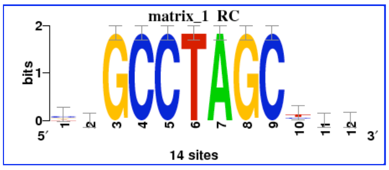

TOMTOM

```
;
; MATRIX 2/2 : matrix_2
;
;
; Matrix type: counts
; Pos   |       1       2       3       4       5       6       7       8       9       10      11
; ----- |-------|-------|-------|-------|-------|-------|-------|-------|-------|-------|-------|
a       |       20      7       34      0       34      0       34      0       34      12      19
c       |       4       5       0       0       0       0       0       0       0       4       3
g       |       4       18      0       34      0       34      0       34      0       16      7
t       |       6       4       0       0       0       0       0       0       0       2       5
; ----- |-------|-------|-------|-------|-------|-------|-------|-------|-------|-------|-------|
; c.sum |       34      34      34      34      34      34      34      34      34      34      34      374
; c.max |       20      18      34      34      34      34      34      34      34      16      19      34
; c.min |       4       4       0       0       0       0       0       0       0       2       3       0
//
; consensus     agAGAGAGAra
; consensus.rc  TYTCTCTCTCT
```

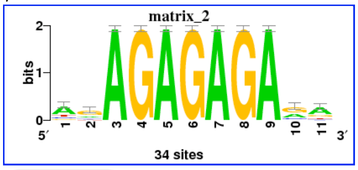

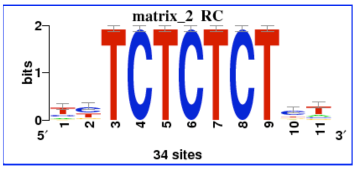

TOMTOM

```
; Job started   2010_07_23.100040
; Job done      2010_07_23.100044
; Seconds       0.32
```

Next step

new

pattern matching (matrix-scan) | convert-matrix | TOMTOM

Compare a single matrix to a motif database.

Warning

```
The matrix      2       /home/rsat/rsa-tools/public_html/tmp/convert-matrix.2010_07_23.100039.res_2     contains 0 columns.
```

Warning

The matrix 4 /home/rsat/rsa-tools/public_html/tmp/convert-matrix.2010_07_23.100039.res_4 contains 0 columns.

---

a |	4	4	7	0	0	0	14	0	0	0	4	3
c |	5	4	1	0	14	0	0	0	0	14	2	3
g |	1	3	4	14	0	0	0	14	14	0	2	7
t |	4	3	2	0	0	14	0	0	0	0	6	1

RSAT-SFY111

RSA-tools - convert-matrix result

```
command: /home/rsat/rsa-tools/perl-scripts/convert-matrix  -v 1 -i /home/rsat/rsa-tools/public_html/tmp/convert-matrix.2010_07_23.100457.input -pseudo 1 -de
```

Result

```
; convert-matrix  -v 1 -i /home/rsat/rsa-tools/public_html/tmp/convert-matrix.2010_07_23.100457.input -pseudo 1 -decimals 1 -perm 0 -from transfac -bg_pseuc
; Input files
;       input   /home/rsat/rsa-tools/public_html/tmp/convert-matrix.2010_07_23.100457.input
; Input format          transfac
; Output files
;       output  /home/rsat/rsa-tools/public_html/tmp/convert-matrix.2010_07_23.100457.res
; Output format         tab
; pseudo-weight         1
;
; MATRIX 1/4 : matrix_2
;
;
; Matrix type: counts
; Pos   |       1       2       3       4       5       6       7       8       9       10      11      12      13      14      15      16      17      18
; ----- |-------|-------|-------|-------|-------|-------|-------|-------|-------|-------|-------|-------|-------|-------|-------|-------|-------|-------|
a       |       35      36      16      20      31      46      16      0       10      19      18      0       11      1       52      15      30      38
c       |       16      11      8       10      0       0       0       0       0       0       0       0       0       1       3       7       22      16
g       |       37      32      33      46      66      67      98      115     105     87      79      100     90      109     11      24      42      37
t       |       27      36      58      39      18      2       1       0       0       9       18      15      14      4       49      69      21      24
; ----- |-------|-------|-------|-------|-------|-------|-------|-------|-------|-------|-------|-------|-------|-------|-------|-------|-------|-------|
; c.sum |       115     115     115     115     115     115     115     115     115     115     115     115     115     115     115     115     115     115
; c.max |       37      36      58      46      66      67      98      115     105     87      79      100     90      109     52      69      42      38
; c.min |       16      11      8       10      0       0       0       0       0       0       0       0       0       1       3       7       21      16
//
; consensus     rdkkrrGGGGGGGGwtrr
; consensus.rc  YYAWCCCCCCCCYYMMHY
```

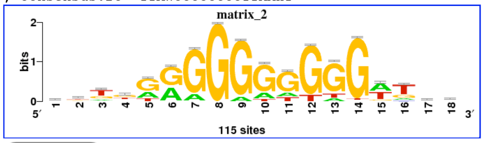

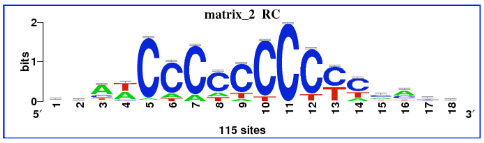

TOMTOM

```
;
; MATRIX 2/4 : matrix_3
;
;
; Matrix type: counts
; Pos   |       1       2       3       4       5       6       7       8       9       10      11
; ----- |-------|-------|-------|-------|-------|-------|-------|-------|-------|-------|-------|
a       |       52      48      0       0       0       34      0       82      0       80      56
c       |       156     170     314     314     314     280     314     232     314     143     141
g       |       39      20      0       0       0       0       0       0       0       23      38
t       |       67      76      0       0       0       0       0       0       0       68      79
; ----- |-------|-------|-------|-------|-------|-------|-------|-------|-------|-------|-------|
; c.sum |       314     314     314     314     314     314     314     314     314     314     314     3454
; c.max |       156     170     314     314     314     280     314     232     314     143     141     314
; c.min |       39      20      0       0       0       0       0       0       0       23      38      0
//
; consensus     ccCCCCCMCmy
; consensus.rc  RKGKGGGGGGG
```

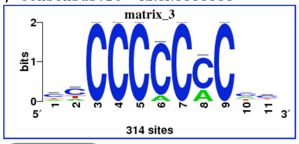

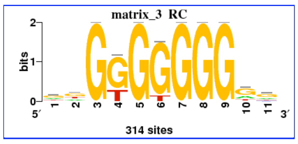

TOMTOM

```
;
; MATRIX 3/4 : matrix_4
;
;
; Matrix type: counts
; Pos   |       1       2       3       4       5       6       7       8       9       10      11      12      13      14      15
; ----- |-------|-------|-------|-------|-------|-------|-------|-------|-------|-------|-------|-------|-------|-------|-------|
a       |       24      31      21      1       44      0       8       0       7       0       6       1       0       25      24
c       |       49      54      98      98      12      93      108     125     118     112     83      63      122     61      62
g       |       24      13      1       2       5       4       0       0       0       0       0       0       0       14      12
t       |       28      27      5       24      64      28      9       0       0       13      36      61      3       25      27
; ----- |-------|-------|-------|-------|-------|-------|-------|-------|-------|-------|-------|-------|-------|-------|-------|
; c.sum |       125     125     125     125     125     125     125     125     125     125     125     125     125     125     125     1875
; c.max |       49      54      98      98      64      93      108     125     118     112     83      63      122     61      62      125
; c.min |       24      13      1       1       5       0       0       0       0       0       0       0       0       14      12      0
//
; consensus     ccCCwCCCCCyyCcc
; consensus.rc  GGGRRGGGGGWGGGG
```

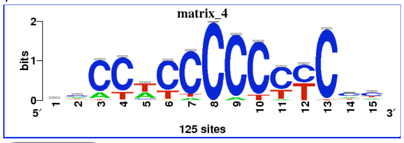

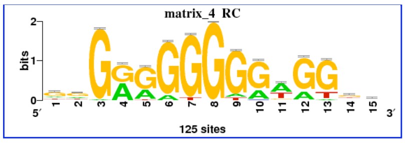

TOMTOM

```
;
; MATRIX 4/4 : matrix_5
;
;
; Matrix type: counts
; Pos   |       1       2       3       4       5       6       7       8       9       10      11      12      13      14      15      16      17      18
; ----- |-------|-------|-------|-------|-------|-------|-------|-------|-------|-------|-------|-------|-------|-------|-------|-------|-------|-------|
a       |       28      28      70      54      3       13      17      15      8       1       0       1       2       3       41      10      41      27
c       |       48      51      41      15      126     108     120     94      104     127     140     123     95      90      85      109     51      55
g       |       24      23      15      1       1       1       0       3       0       0       0       1       0       0       11      5       11      19
t       |       40      38      14      70      10      18      3       28      28      12      0       15      43      47      3       16      37      39
```

; -----	--------	-------	-------	-------	-------	-------	-------	-------	-------	-------	-------	-------	-------	-------	-------	-------	-------	-------
; c.sum	140	140	140	140	140	140	140	140	140	140	140	140	140	140	140	140	140	140
; c.max	48	51	70	70	126	108	120	94	104	127	140	123	95	90	85	109	51	55
; c.min	24	23	14	1	1	1	0	3	0	0	0	1	0	0	3	5	11	19

```
//
; consensus      yymwCCCcCCCCyymChy
; consensus.rc   RDGKRRGGGGGGGGWKRR
```

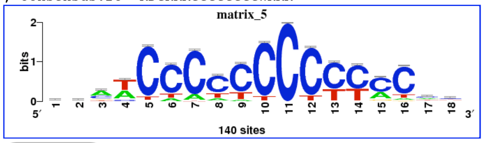

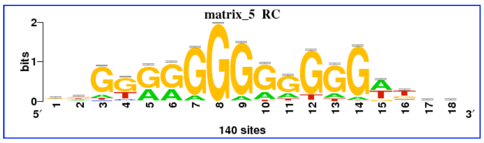

TOMTOM

```
; Job started    2010_07_23.100457
; Job done       2010_07_23.100506
; Seconds        0.36
```

Next step

new

pattern matching (matrix-scan) convert-matrix

TOMTOM

Compare a single matrix to a motif database.

Warning

The matrix 2 /home/rsat/rsa-tools/public_html/tmp/convert-matrix.2010_07_23.100457.res_2 contains 0 columns.

Warning

The matrix 4 /home/rsat/rsa-tools/public_html/tmp/convert-matrix.2010_07_23.100457.res_4 contains 0 columns.

Warning

The matrix 6 /home/rsat/rsa-tools/public_html/tmp/convert-matrix.2010_07_23.100457.res_6 contains 0 columns.

Warning

The matrix 8 /home/rsat/rsa-tools/public_html/tmp/convert-matrix.2010_07_23.100457.res_8 contains 0 columns.

---

a	35	36	16	20	31	46	16	0	10	19	18	0	11	1	52	15	30	38
c	16	11	8	10	0	0	0	0	0	0	0	0	0	1	3	7	22	16
g	37	32	33	46	66	67	98	115	105	87	79	100	90	109	11	24	42	37
t	27	36	58	39	18	2	1	0	0	9	18	15	14	4	49	69	21	24

RSAT-SFY115

RSA-tools - convert-matrix result

```
command: /home/rsat/rsa-tools/perl-scripts/convert-matrix  -v 1 -i /home/rsat/rsa-tools/public_html/tmp/convert-matrix.2010_07_23
```

Result

```
; convert-matrix  -v 1 -i /home/rsat/rsa-tools/public_html/tmp/convert-matrix.2010_07_23.100729.input -pseudo 1 -decimals 1 -perm
; Input files
;       input   /home/rsat/rsa-tools/public_html/tmp/convert-matrix.2010_07_23.100729.input
; Input format          transfac
; Output files
;       output  /home/rsat/rsa-tools/public_html/tmp/convert-matrix.2010_07_23.100729.res
; Output format         tab
; pseudo-weight         1
;
; MATRIX 1/3 : matrix_1
;
;
; Matrix type: counts
; Pos   |       1       2       3       4       5       6       7       8       9       10      11      12
; ----- |-------|-------|-------|-------|-------|-------|-------|-------|-------|-------|-------|-------|
a       |       3       6       0       0       0       0       12      0       0       0       4       4
c       |       4       2       0       5       12      0       0       0       7       12      0       1
g       |       1       0       12      7       0       0       0       12      5       0       2       4
t       |       4       4       0       0       0       12      0       0       0       0       6       3
; ----- |-------|-------|-------|-------|-------|-------|-------|-------|-------|-------|-------|-------|
; c.sum |       12      12      12      12      12      12      12      12      12      12      12      12      144
; c.max |       4       6       12      7       12      12      12      12      7       12      6       4       12
; c.min |       1       0       0       0       0       0       0       0       0       0       0       1       0
//
; consensus     hwGsCTAGsCwd
; consensus.rc  HWGSCTAGSCWD
```

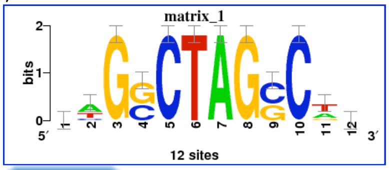

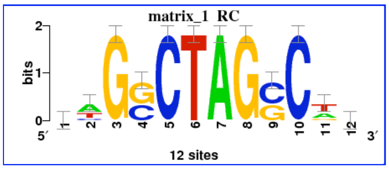

TOMTOM

```
;
; MATRIX 2/3 : matrix_2
;
;
; Matrix type: counts
; Pos   |       1       2       3       4       5       6       7       8       9       10      11
; ----- |-------|-------|-------|-------|-------|-------|-------|-------|-------|-------|-------|
a       |       3       1       0       0       11      0       0       0       11      10      3
c       |       0       1       11      11      0       11      0       0       0       0       3
g       |       3       1       0       0       0       0       11      11      0       0       0
t       |       5       8       0       0       0       0       0       0       0       1       5
; ----- |-------|-------|-------|-------|-------|-------|-------|-------|-------|-------|-------|
; c.sum |       11      11      11      11      11      11      11      11      11      11      11      121
; c.max |       5       8       11      11      11      11      11      11      11      10      5       11
; c.min |       0       1       0       0       0       0       0       0       0       0       0       0
//
; consensus     dTCCACGGAAh
; consensus.rc  DTTCCGTGGAH
```

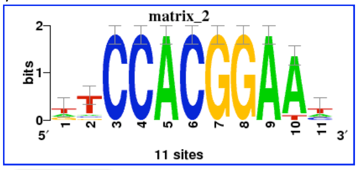

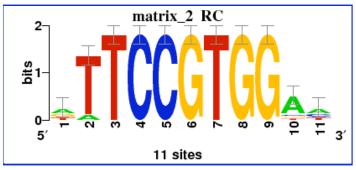

TOMTOM

```
;
; MATRIX 3/3 : matrix_3
;
;
; Matrix type: counts
; Pos   |       1       2       3       4       5       6       7       8       9       10      11      12
; ----- |-------|-------|-------|-------|-------|-------|-------|-------|-------|-------|-------|-------|
a       |       0       1       0       0       0       0       0       0       0       0       1       0
c       |       2       0       0       0       2       2       0       0       2       2       0       0
g       |       0       0       2       2       0       0       2       2       0       0       0       2
t       |       0       1       0       0       0       0       0       0       0       0       1       0
; ----- |-------|-------|-------|-------|-------|-------|-------|-------|-------|-------|-------|-------|
; c.sum |       2       2       2       2       2       2       2       2       2       2       2       2       24
; c.max |       2       1       2       2       2       2       2       2       2       2       1       2       2
; c.min |       0       0       0       0       0       0       0       0       0       0       0       0       0
//
; consensus     CwGGCCGGCCwG
; consensus.rc  CWGGCCGGCCWG
```

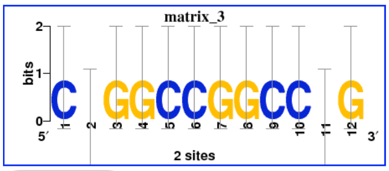

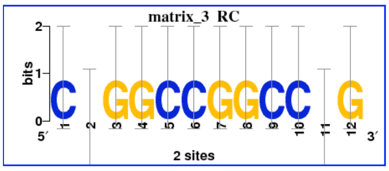

TOMTOM

```
; Job started    2010_07_23.100730
; Job done       2010_07_23.100736
; Seconds        0.31
```

Next step

new

pattern matching (matrix-scan) | convert-matrix | TOMTOM

Compare a single matrix to a motif database.

Warning

The matrix 2 /home/rsat/rsa-tools/public_html/tmp/convert-matrix.2010_07_23.100729.res_2 contains 0 columns.

Warning

The matrix 4 /home/rsat/rsa-tools/public_html/tmp/convert-matrix.2010_07_23.100729.res_4 contains 0 columns.

Warning

The matrix 6 /home/rsat/rsa-tools/public_html/tmp/convert-matrix.2010_07_23.100729.res_6 contains 0 columns.

---

a	|	3	6	0	0	0	0	12	0	0	0	4	4
c	|	4	2	0	5	12	0	0	0	7	12	0	1
g	|	1	0	12	7	0	0	0	12	5	0	2	4
t	|	4	4	0	0	0	12	0	0	0	0	6	3

RSAT-SFY116

RSA-tools - convert-matrix result

```
command: /home/rsat/rsa-tools/perl-scripts/convert-matrix  -v 1 -i /home/rsat/rsa-tools/public_html/tmp/convert-matrix.2010_07_23
```

Result

```
; convert-matrix  -v 1 -i /home/rsat/rsa-tools/public_html/tmp/convert-matrix.2010_07_23.101027.input -pseudo 1 -decimals 1 -perm
; Input files
;       input   /home/rsat/rsa-tools/public_html/tmp/convert-matrix.2010_07_23.101027.input
; Input format          transfac
; Output files
;       output  /home/rsat/rsa-tools/public_html/tmp/convert-matrix.2010_07_23.101027.res
; Output format         tab
; pseudo-weight         1
;
; MATRIX 1/2 : matrix_2
;
;
; Matrix type: counts
; Pos   |       1       2       3       4       5       6       7       8       9       10      11      12      13
; ----- |-------|-------|-------|-------|-------|-------|-------|-------|-------|-------|-------|-------|-------|
a       |       2       0       0       0       9       0       0       0       9       9       2       2       2
c       |       0       1       9       9       0       9       0       0       0       0       2       4       0
g       |       2       1       0       0       0       0       9       9       0       0       0       2       4
t       |       5       7       0       0       0       0       0       0       0       0       5       1       3
; ----- |-------|-------|-------|-------|-------|-------|-------|-------|-------|-------|-------|-------|-------|
; c.sum |       9       9       9       9       9       9       9       9       9       9       9       9       9       117
; c.max |       5       7       9       9       9       9       9       9       9       9       5       4       4       9
; c.min |       0       0       0       0       0       0       0       0       0       0       0       1       0       0
//
; consensus     tTCCACGGAAtck
; consensus.rc  MGATTCCGTGGAA
```

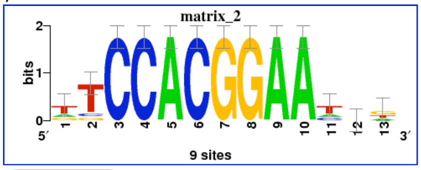

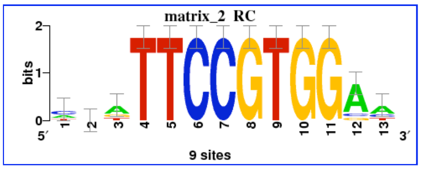

TOMTOM

```
;
; MATRIX 2/2 : matrix_3
;
;
; Matrix type: counts
; Pos   |       1       2       3       4       5       6       7       8       9       10      11      12
; ----- |-------|-------|-------|-------|-------|-------|-------|-------|-------|-------|-------|-------|
a       |       3       1       5       5       0       0       0       0       0       0       4       2
c       |       0       1       0       0       5       5       5       0       0       0       1       1
g       |       1       1       0       0       0       0       0       0       0       0       0       0
t       |       1       2       0       0       0       0       0       5       5       5       0       2
; ----- |-------|-------|-------|-------|-------|-------|-------|-------|-------|-------|-------|-------|
; c.sum |       5       5       5       5       5       5       5       5       5       5       5       5       60
; c.max |       3       2       5       5       5       5       5       5       5       5       4       2       5
; c.min |       0       1       0       0       0       0       0       0       0       0       0       0       0
//
; consensus     atAACCCTTTAw
; consensus.rc  WTAAAGGGTTAT
```

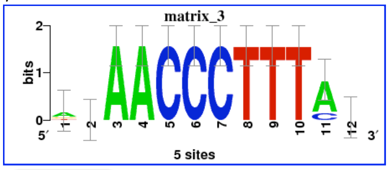

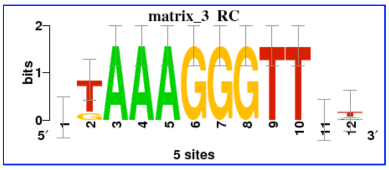

TOMTOM

```
; Job started   2010_07_23.101028
; Job done      2010_07_23.101032
; Seconds       0.28
```

Next step

new

pattern matching (matrix-scan) convert-matrix TOMTOM

Compare a single matrix to a motif database.

Warning

```
The matrix      2       /home/rsat/rsa-tools/public_html/tmp/convert-matrix.2010_07_23.101027.res_2     contains 0 columns.
```

Warning

The matrix 4 /home/rsat/rsa-tools/public_html/tmp/convert-matrix.2010_07_23.101027.res_4 contains 0 columns.

---

a	|	2	0	0	0	9	0	0	0	9	9	2	2	2
c	|	0	1	9	9	0	9	0	0	0	0	2	4	0
g	|	2	1	0	0	0	0	9	9	0	0	0	2	4
t	|	5	7	0	0	0	0	0	0	0	0	5	1	3
